# The GntR/VanR transcription regulator AlkR represses AlkB2 monooxygenase expression and regulates *n*‐alkane degradation in *Pseudomonas aeruginosa* SJTD‐1

**DOI:** 10.1002/mlf2.70004

**Published:** 2025-04-21

**Authors:** Wanli Peng, Xiuli Wang, Qinchen Liu, Zhihong Xiao, Fulin Li, Nannan Ji, Zhuo Chen, Jiaying He, Junhao Wang, Zixin Deng, Shuangjun Lin, Rubing Liang

**Affiliations:** ^1^ State Key Laboratory of Microbial Metabolism, Joint International Research Laboratory of Metabolic & Developmental Sciences, School of Life Sciences & Biotechnology Shanghai Jiao Tong University Shanghai China

**Keywords:** AlkB2 monooxygenase, AlkR transcription regulator, regulatory mechanism, structure features, VanR–AlkB couples

## Abstract

Transmembrane alkane monooxygenase (AlkB)‐type monooxygenases, especially AlkB2 monooxygenases, are crucial for aerobic degradation of the medium‐to‐long‐chain *n*‐alkanes in hydrocarbon‐utilizing microorganisms. In this study, we identified a GntR/VanR transcription regulator AlkR of *Pseudomonas aeruginosa* SJTD‐1 involved in the negative regulation of AlkB2 and deciphered its nature of DNA binding and ligand release. The deletion of *alkR* enhanced the transcription levels of the *alkB2* gene and the utilization efficiency of the medium‐to‐long‐chain *n*‐alkanes by strain SJTD‐1. The dimer of AlkR recognizes and binds to a conserved palindromic motif in the promoter of the *alkB*2 gene, and structural symmetry is vital for DNA binding and transcription repression. The long‐chain fatty acyl coenzyme A compounds can release AlkR and stimulate transcription of *alkB*2, reflecting the effect of alkane catabolic metabolites. Structural insights unveiled that the arginine residues and scaffold residues of AlkR are critical for DNA binding. Further bioinformatics analysis of AlkR revealed the widespread VanR–AlkB couples distributed in *Pseudomonadaceae* with high conservation in the sequences of functional genes and intergenic regions, highlighting a conserved regulatory pattern for *n*‐alkane utilization across this family. These findings demonstrate the regulatory mechanism and structural basis of GntR/VanR transcription regulators in modulating *n*‐alkane biodegradation and provide valuable insights in improving the bioremediation efficiency of hydrocarbon pollution.

## Impact statement

Alkanes are the major components of petroleum hydrocarbon contaminations, and microbial degradation is effective and important for the carbon cycle. Biodegradation efficiency decreases with increasing chain length, and biotransformation of high‐molecular‐weight *n*‐alkanes is difficult and they persistent in the environment for a long time. How do bacteria precisely regulate the enzyme activities and efficiently catalyze *n*‐alkane biodegradation? This study reveals a novel GntR/VanR regulator and a universal regulator–enzyme regulation mode widely present in petroleum‐utilizing bacteria, and elucidates the regulatory mechanism and structural basis. The findings formulate the common regulation pattern of *n*‐alkane biodegradation and offer new insights for hydrocarbon pollution bioremediation.

## INTRODUCTION

With industrial development, annually, over 800 million tons of hydrocarbons are discharged into the environment through different sources, such as oil spills, natural seeps, or accidental release from the petroleum industry^1^. Released petroleum pollutants have become one of the most critical environment challenges, resulting in a significant threat to various ecosystems and inducing irreversible damage to the survival and health of all living beings^2^, ^3^. In petroleum‐polluted environments, organisms, especially microorganisms, have evolved and developed the capability to sense and utilize these hydrocarbons as carbon or energy sources, constituting an essential component of the global carbon cycle. Consequently, microbial biodegradation has been widely regarded as an environmentally friendly and cost‐effective strategy for hydrocarbon removal and environment remediation^1^, ^2^, ^3^. Various microorganisms, including bacteria, fungi, and algae, have been reported to be able to utilize diverse hydrocarbon pollutants as carbon sources; among them, bacteria are considered the primary and efficient mediators^4^, ^5^. Numerous bacterial strains isolated from different ecosystems have been identified with remarkable efficiency in petroleum degradation, such as strains of *Pseudomonas*, *Acinetobacter*, and *Rhodococcus* ^6^, ^7^, ^8^, ^9^, ^10^.

In petroleum‐polluted environments, bacteria may encounter a spectrum of *n*‐alkanes, the predominant fraction of petroleum pollutants, categorized into short‐chain (C1–C5), medium‐chain (C6–C12), medium‐to‐long chain (C14–C20), and long‐chain (>C20) *n*‐alkanes. The biodegradation process is more difficult for medium‐to‐long chain and long‐chain *n*‐alkanes relative to that of short‐chain *n*‐alkanes, posing a major challenge^4^, ^11^. In most aerobic bacteria, *n*‐alkanes can be sequentially transformed into alcohols, aldehydes, and fatty acids, followed by conjugation with coenzyme A and entry into β‐oxidation pathways for energy acquisition^12^. The initial and rate‐limited step is the oxidation of terminal methyl groups, catalyzed by three classes of monooxygenases^4^, ^13^, ^14^, ^15^, ^16^. Bacteria typically harbor multiple copies and diverse types of hydroxylases with overlapping substrate spectra to achieve effective utilization of *n*‐alkanes of different lengths^13^, ^16^, ^17^, ^18^. Among them, transmembrane alkane monooxygenase (AlkB) plays a predominant role in oxidizing a wide range of *n*‐alkanes of different chain lengths, mainly liquid *n*‐alkanes (C5–C20)^4^, ^18^, ^19^, ^20^, ^21^. AlkB monooxygenases are classified within the membrane fatty acid desaturase (FADS)‐like superfamily, representing a class of non‐heme diiron monooxygenases for the desaturation or hydroxylation of fatty acyl aliphatic chains, ubiquitous across bacterial species and over 6,000 AlkBs in the NCBI protein database^21^, ^22^. Consequently, AlkBs are recognized as the principal catalysts for *n*‐alkane oxidation in petroleum‐degrading bacteria^15^, ^23^, ^24^. Therefore, AlkB1 monooxygenase specializes in the hydroxylation of medium‐chain *n*‐alkanes (C6–C12), and AlkB2 monooxygenase predominantly catalyzes oxidation of medium‐to‐long chain *n*‐alkanes (C14–C20)^17^, ^18^, ^21^, ^22^. In addition, the soluble flavoprotein alkane monooxygenase LadA and the flavin‐binding monooxygenase AlmA are implicated in the oxidation of long‐chain *n*‐alkanes^10^, ^17^, ^25^, ^26^. In *P. aeruginosa* SJTD‐1, an efficient petroleum degrader of medium‐to‐long chain *n*‐alkanes (C12–C32), there are two AlkB monooxygenases (AlkB1 and AlkB2), two LadA hydroxylases (LadA1 and LadA2), and two AlmA‐like monooxygenases (AlmA1 and AlmA2) that contribute to the alkane‐utilizing capacities of this strain.

Moreover, to maintain robust and specific enzymatic activities, a precise regulatory network is necessary for adept and efficient handling of *n*‐alkanes in bacteria^27^, ^28^, ^29^, ^30^. Several transcriptional regulators, such as AraC/XylS, LuxR/MalT, TetR, AlmR, and CrgA, have been reported to be involved in the regulation of *n*‐alkane degradation by modulating the transcription of AlkB monooxygenases^29^, ^31^, ^32^, ^33^. AlmR repressed the expression of AlmA monooxygenase and regulated long‐chain alkane metabolism in *A. dieselolei* ^32^. CypR (AraC family) and AlkX (TetR family) could activate and repress the alkane hydroxylases CYP153 and AlkW1 in *Dietzia* sp.^33^, ^34^. The expression of AlkM in *Acinetobacter* sp. ADP1 was regulated by AlkR, a member of the AraC/XylS family^35^. The global regulator CrgA in *P. aeruginosa* SJTD‐1 acted as a negative modulator of AlkB2 and participated in the regulation of *n*‐alkane metabolism^27^, ^32^, ^34^. Considering that diverse enzymes are responsible for the catalysis of *n*‐alkanes of different lengths, there must exist specific transcriptional regulators and differential regulatory modes for precise control and efficient utilization in petroleum‐degrading bacteria. However, even to the most common AlkB‐type monooxygenases, the transcription regulators and regulatory mechanisms have yet to be clearly elucidated, leaving a gap in this critical metabolic pathway.

We have previously reported that *P. aeruginosa* SJTD‐1 efficiently degrades medium‐ and long‐chain *n*‐alkanes, and the cellular metabolism is changed for the response and utilization of *n*‐alkanes. AlkB2 monooxygenase is the main catalyst of medium‐to‐long‐chain *n*‐alkanes, negatively regulated by a distant regulator CrgA^8^, ^21^, ^29^, ^36^, ^37^. In this work, we identified a novel transcription regulator AlkR in *P. aeruginosa* SJTD‐1, determined its characteristics of DNA binding and release, and elucidated structural insights as a specific repressor of AlkB2 monooxygenase. This is the first report of the participation of GntR/VanR transcriptional regulators in the regulation of *n*‐alkane biodegradation. We also revealed a universal and conserved distribution of VanR–AlkB2 regulatory–enzyme couples across diverse taxonomic groups, implying a common regulatory mode for effective *n*‐alkane metabolism in bacteria.

## RESULTS

### AlkR tightly represses the transcription of *alkB*2 and regulates the utilization of *n*‐alkanes in *P. aeruginosa* SJTD‐1

AlkB2 monooxygenase is pivotal for the degradation of *n*‐alkanes (C14‐C20) in *P. aeruginosa* SJTD‐1, and the transcription of *alkB*2 can be induced by medium‐to‐long‐chain *n*‐alkanes^21^. In the upstream of *alkB*2 gene, there is one gene (*A214_15800*) in the reverse direction that encodes a transcription regulator of the GntR superfamily, implying that it probably regulates the expression of AlkB2 monooxygenase. To determine whether this regulator directly controls the transcription of the *alkB*2 gene and identify the potential regulators of AlkB2, the pull‐down assay was performed with the immobilized upstream fragment of the *alkB2* gene and the crude cell lysates of strain SJTD‐1 cultured with *n*‐octadecane (C18). The mass spectrometry analysis results showed that the enriched protein (ANI09869.1), containing 219 amino acid residues, is a member of the FCD subfamily (FadR C‐terminal domain, Pfam ID PF07729) belonging to GntR superfamily transcription regulators, named as AlkR and encoded by gene *A214_15800* (*alkR*) (Figure S1A). Another enriched protein is the global transcription regulator CrgA that we have found to be involved in the regulation of AlkB2 before^36^. Thus, the transcription regulator AlkR is likely to modulate the transcription of AlkB2. The recombinant AlkR protein was obtained by heterologous expression and affinity purification. A chemical cross‐linking assay with sulfo‐EGS showed that AlkR protein is predominantly in the dimer form in solution, consistent with the results of size exclusion chromatography (Figure S1B,C).

**Figure 1:**
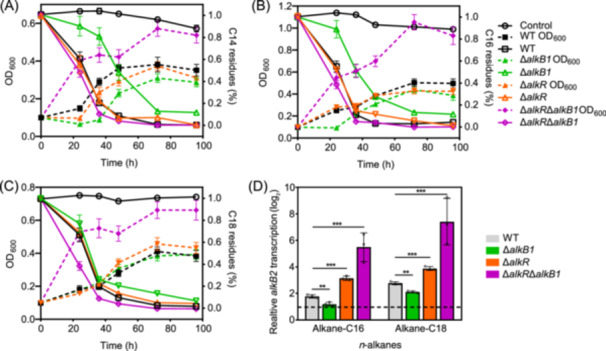
AlkR regulates the alkane‐degrading efficiency of *P. aeruginosa* SJTD‐1. The alkane‐degrading efficiency and the cell growth rate of wild‐type SJTD‐1 (WT) and mutant strains (∆*alkB1*, ∆*alkR*, ∆*alkR*∆*alkB1*) were assessed in minimal medium containing 500 mg/l *n*‐alkanes (C14, C16, or C18) as the sole carbon source. (A) Curves of the alkane‐degrading efficiency and the cell growth rate of these strains cultured in C14‐alkane. (B) Curves of the alkane‐degrading efficiency and the cell growth rate of these strains cultured in C16‐alkane. (C) Curves of the alkane‐degrading efficiency and the cell growth rate of these strains cultured in C18‐alkane. (D) Transcription levels of the *alkB2* gene in these strains in response to *n*‐alkanes (C16 and C18). Strains were cultured for 24 h, with the wild‐type strain cultured in minimal medium with 2% glucose as a control. Three independent experiments were performed and the average values were presented with the statistical analysis (*n* = 3; mean ± SD; *p < 0.05; **p < 0.01; ***p < 0.001). C14, *n*‐tetradecane; C16, *n*‐hexadecane; C18, *n*‐octadecane.

To investigate the regulatory role of AlkR in the transcription of the *alkB2* gene, single‐gene knockout mutant strain ∆*alkR, ∆alkB1,* and multiple‐gene knockout mutant ∆*alkB1*∆*alkR* of strain SJTD‐1 were constructed; their ability to utilize *n*‐alkanes *n*‐tetradecane (C14), *n*‐hexadecane (C16), or *n*‐octadecane (C18) as sole carbon sources was assessed. The results indicated that strain ∆*alkR* showed comparable cell growth rate and alkane‐degrading efficiency to those of strain SJTD‐1, while strain ∆*alkB1* showed slower growth rate and reduced degradation capacity. However, in the culture of C18, strain ∆*alkB1* displayed similar alkane‐degrading efficiency and growth tendency to those of strain SJTD‐1 and strain ∆*alkR* (Figure 1A–C). This is consistent with previous results showing that AlkB1 monooxygenase mainly plays a role in degrading medium‐chain *n*‐alkanes, and there are still other regulators like CrgA that regulate AlkB2 monooxygenase^21^. Notably, the double‐knockout strain ∆*alkR*∆*alkB1* displayed a significant increase in both cell growth and degradation efficiency using the three *n*‐alkanes, particularly with C18. The biomass accumulation and the alkane‐degrading efficiency of strain ∆*alkR*∆*alkB1* after 24 h were 3–4‐fold and 10%–20% higher, respectively, than those of strain SJTD‐1 (Figure 1A–C). These results demonstrated that AlkR plays a major role in the negative and specific transcriptional regulation of the *alkB2* gene, and the addition of C18 contributes toward alleviating the inhibitory effect of AlkR.

Moreover, the transcription of the *alkB2* gene in strain SJTD‐1 and the mutant strains cultured with glucose, C16, or C18 was analyzed. The transcription levels of the *alkB2* gene in all these strains cultured with C16 or C18 were upregulated compared to those in the culture with glucose, and the induction of C18 was more remarkable. The significant increase was observed in the *alkR*‐deleted strains (strain ∆*alkR*∆*alkB1* and strain ∆*alkR*) cultured with C18 (Figure 1D). Extra deletion of the *crgA* gene (strain ∆*alkR*∆*alkB1*∆*crgA*) enhanced the transcription level of the *alkB2* gene slightly. Conversely, overexpression of AlkR in strain ∆*alkR*∆*alkB1*∆*crgA* resulted in a significant decrease in the transcription levels of the *alkB2* gene, both in cultures with C16 and C18 (Figure S2). Further, the impact of AlkR on the transcription of the *alkB2* gene was assessed using the reporter plasmids, with or without the *alkR* gene in the upstream of the *alkB2* promoter and the *eGFP* gene, and these plasmids were transformed into strain SJTD‐1 and strain ∆*alkR* (Figure S3). The results showed that the expression of eGFP protein in all the transformants was induced by three medium‐to‐long‐chain *n*‐alkanes (C14–C18), but considerably by C18. Strain ∆*alkR* showed significantly higher fluorescence intensities than those of strain SJTD‐1 when cultured in three carbon sources of different lengths. In contrast, overexpression of AlkR in strain SJTD‐1 and strain ∆*alkR* both led to a noteworthy decrease in the fluorescence intensities, significantly in the culture with C18 (Figure S3). This suggests that AlkR tightly suppresses the transcription of the *alkB2* gene, which can be released by medium‐to‐long chain *n*‐alkanes to facilitate *n*‐alkane utilization.

### The long‐chain fatty acyl‐CoA compounds release AlkR from its specific binding site in the upstream of the *alkB*2 gene

**Figure 2:**
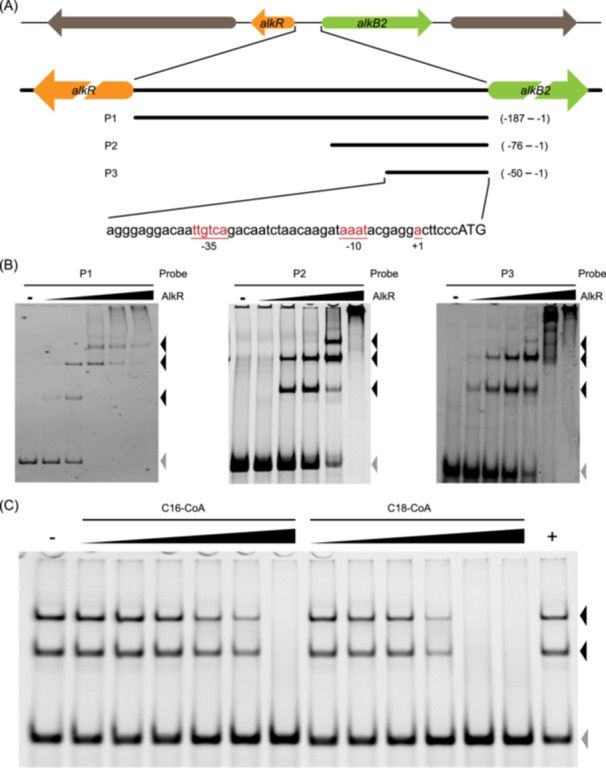
The AlkR protein can efficiently bind to the promoter region of the *alkB*2 gene in vitro and the binding can be released by two long‐chain fatty acyl‐CoA compounds. (A) Schematic diagram of the location and the intergenic region of *alkB2* and *alkR* genes. The ‒10 and ‒35 regions of the *alkB2* promoter and the transcriptional start site (+1) of the *alkB2* gene are highlighted. The three DNA fragments of different lengths in the *alkB*2 promoter (P1‐P3) are shown, ranging from positions –50 bp, –76 bp, and –187 bp to the start codon ATG of the *alkB*2 gene. (B) Recombinant AlkR could bind to the three DNA fragments (P1, P2, P3) in a concentration‐dependent manner. The EMSA assays were performed at various AlkR/DNA molar ratios, with AlkR/P1 (0.2, 1.0, 2.0, 10.0, 20.0, from left to right), AlkR/P2 (0.1, 0.2, 1.0, 5.0, 10.0, from left to right), and AlkR/P3 (0.2, 1.0, 2.0, 5.0, 10.0, 20.0, from left to right). The BSA protein was used as a negative control (–). The gray triangles represent the shift bands of unbound DNA fragments, and the black triangles represent different shift bands of the protein‐bound DNA fragments. (C) Two long‐chain fatty acyl‐CoA compounds (C16‐CoA and C18‐CoA) could release the binding of AlkR to the P3 fragment. Two long‐chain fatty acyl‐CoA (C16‐CoA or C18‐CoA, 5.0, 10.0, 20.0, 40.0, 80.0, and 150.0 μM, from left to right) were added to the binding system with AlkR (5.8 × 10^−7^ M) and P3 fragments (5 × 10^−7^ M), and then assessed by EMSA. The reactions supplied without an effector or supplied with acetyl‐CoA (600 μM) were used as a negative control (–) and a positive control (+), respectively. The profiles of 12% PAGE from three independent assays are shown. The gray triangles represent the shift bands of unbound DNA fragments and the black triangles represent different shift bands of the protein‐bound DNA fragments.

To analyze the binding properties of AlkR protein to the promoter region of the *alkB2* gene, electrophoretic mobility shift assay (EMSA) assays were conducted with AlkR and three fragments of the *alkB2* promoter (P1‐P3) with different lengths, ranging from positions –50, –76, and –187 bp to the start codon ATG of the *alkB2* gene, respectively (Figure 2A). The results showed that the recombinant AlkR protein could bind to all three fragments efficiently in vitro in a dose‐dependent manner, and effective binding was observed at a protein‐to‐DNA ratio of 0.2 (Figure 2B). This demonstrates that AlkR binds to the promoter of the *alkB2* gene with high affinity and specificity, and its critical binding site is within the 50 bp upstream of the *alkB2* gene. In addition, in strain SJTD‐1, there still exist two genes *ladA1* and *almA*, which have been reported to be involved in the utilization of the long‐chain *n*‐alkanes^21^. Here, similar binding was also observed between the recombinant AlkR protein and the fragments from the promoters of *ladA1* and *almA* genes, accompanied by much weaker binding of AlkR to the fragment from the promoter of the *ladA2* gene, implying the distant regulatory role of AlkR in these catalysts for utilizing long‐chain *n*‐alkanes (Figure S4).

As the transcription of the *alkB*2 gene can be induced by *n*‐alkanes, these chemicals or their derivants are likely to be effectors of AlkR for its release from DNA. The effects of *n*‐alkane substrates and their metabolic derivatives on the interaction of the AlkR/DNA complex were evaluated. Notably, the robust binding of AlkR to the promoter of the *alkB2* gene was effectively dissociated only by two long‐chain fatty acyl‐CoA compounds, palmitoyl coenzyme A (C16‐CoA) and oleyl coenzyme A (C18‐CoA), with the concentration being approximately 150 μM for C16‐CoA and 80 μM for C18‐CoA (Figure 2C). In contrast, negligible effects on the AlkR/DNA complex were observed using acetyl coenzyme A, *n*‐alkanes (C16 and C18), their corresponding fatty alcohols (hexadecanol (C16‐OH) and octadecanol (C18‐OH)) or fatty acids (*n‐*hexadecanoic acid (C16‐COOH), *n*‐octadecanoic acid (C18‐COOH), and sodium *n*‐octadecanoate (C18‐COONa)), even at elevated concentrations of up to 600 μM (Figure S5). Moreover, the binding of AlkR to the promoter regions of *ladA1* and *almA* genes was similarly released by C16‐CoA and C18‐CoA (Figure S4). This means that the end oxidized intermediate of medium‐to‐long‐chain *n*‐alkanes, before β‐oxidation, serve as valid effectors for AlkR protein release and transcription initiation of the *alkB2* gene. AlkR, in conjunction with long‐chain fatty acyl‐CoA compounds, constitutes a sophisticated regulatory circuitry involving negative control and positive feedback for AlkB2 monooxygenase, ensuring responsive and effective metabolic adaptation to available *n*‐alkane substrates.

### A conserved palindromic motif is critical for AlkR recognition and binding

**Figure 3:**
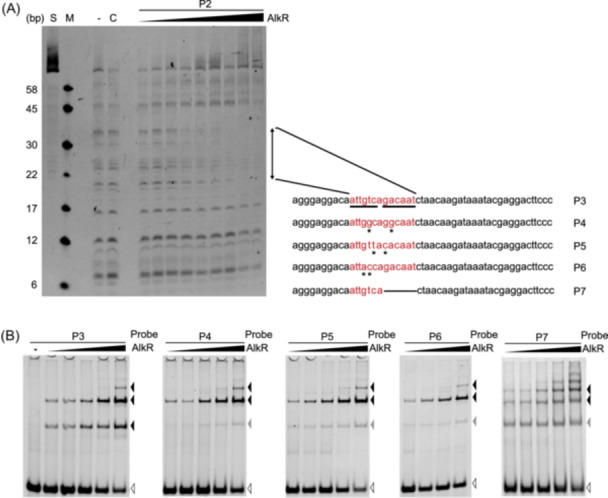
The AlkR protein binds to the specific motif in the promoter of the *alkB2* gene. (A) The DNase I footprinting assay showed that AlkR could protect the specific region of the promoter of the *alkB2* gene. The 5′‐FAM‐labeled DNA fragment P2 was the target DNA fragment, which was incubated with or without AlkR and then digested with DNase I. The lanes of the P2 fragment without the treatment of DNase I (S), DNA marker (M), the P2 fragment treated by DNase I (−), and the P2 fragment mixed with BSA treated by DNase I (C) are labeled. The protected region in the P2 fragment corresponding to the P3 DNA fragment is highlighted in red. Fragments P4/P5/P6/P7 were the mutant DNA fragments from the fragment P3. The underline dashes in fragment P3 present the two modified regions, and the dash in fragment P7 indicates this region was deleted. (B) The EMSA analysis showed the varied binding properties of AlkR to the mutant DNA fragments. The binding of AlkR protein to the mutant DNA fragments was detected by EMSA. The DNA fragments (5 × 10^−7^ M) were incubated with AlkR protein in increasing concentrations (from left to right, for fragments P3/P4/P5/P7: 7.5 × 10^−8 ^M, 1.5 × 10^−7 ^M, 3 × 10^−7 ^M, 6 × 10^−7 ^M, and 1.2 × 10^−6 ^M; for fragment P6: 7.5 × 10^−8^ M, 1.5 × 10^−7 ^M, 3 × 10^−7 ^M and 6 × 10^−7 ^M). The bands of the DNA fragments (white triangle), the shifted bands (black triangle), and the weakened bands (gray triangle) are labeled. The 12% native polyacrylamide gel was used for the analyses. The white triangles represent the shift bands of unbound DNA fragments. The black triangles represent the shift bands of the protein‐bound DNA fragments (P4/P5/P6/P7) same as those of the protein‐bound P3 fragment. The gray triangles represent the new shift bands of the protein‐bound DNA fragments (P4/P5/P6/P7).

The precise binding site of AlkR within the promoter region of the *alkB*2 gene was determined with the DNase I foot‐printing assay using AlkR protein and DNA fragment P2. The results showed that there existed only one protected site (5′‐ATTGTCAGACAAT‐3′) spanning from positions –27 to –40 bp relative to the ATG start codon of the *alkB2* gene. It was considered as the specific binding region of AlkR protein (Figure 3A). This motif for AlkR binding adopts a palindromic sequence structure, similar to the conserved DNA binding motif of FCD subfamily regulators.

To further elucidate the critical sites within the AlkR‐binding motif, several site‐mutant DNA fragments (P4, P5, P6) derived from the wild‐type DNA fragment (P3) were generated, and their binding affinities to AlkR protein were detected. When alterations in any two sites in the symmetrical core sequences (5′‐GTCAGAC‐3′) were introduced (P4, P5, and P6 fragments), the primary binding shift band disappeared and the super‐shift bands remained stable (Figure 3B). This suggested that the 7‐bp sites are crucial for DNA binding of AlkR, and their mutations change the binding properties. Moreover, when the fragment with only half of the 7‐bp binding motif (P7 fragment) was used, two extra binding bands of AlkR emerged in addition to those of the wild‐type P3 fragment (Figure 3B). This indicated that the complete palindromic structure in the promoter of the *alkB2* gene is crucial for the valid and specific binding of AlkR protein. Interestingly, the super‐shift bands were still observed in the binding of AlkR to both the wild‐type fragment P3 and the mutant fragments P4, P5, and P6 (Figure 3B). This implies that the mutations at certain sites have little effect on the super‐binding capacity of AlkR to the promoter of the *alkB2* gene. This also raises the possibility that there may be more than one binding site recognized by different oligomeric forms of AlkR protein.

**Figure 4:**
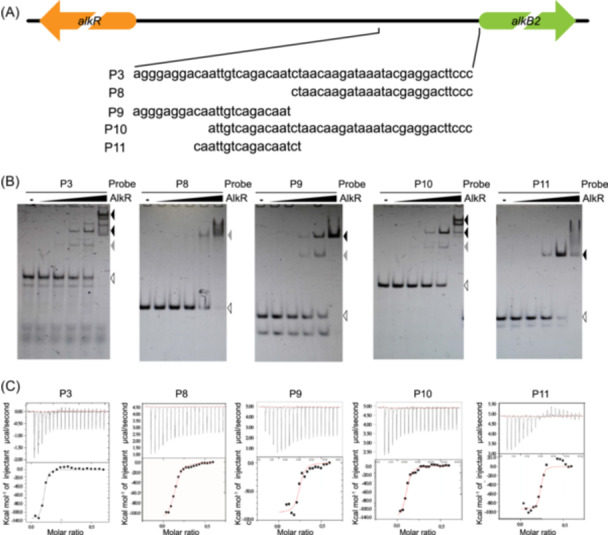
The binding properties of AlkR protein to different fragments of the *alkB2* promoter are diverse. (A) Schematic diagram of DNA fragments (P3/P8/P9/P10/P11) used for isothermal titration calorimetry (ITC) analysis. (B) The binding affinities of AlkR to these DNA fragments are different in EMSA analysis. The DNA fragment bands are labeled with a white triangle, the proposed homomultimeric AlkR–DNA complexes are labeled with a black triangle, and the proposed dimer or tetramer AlkR‐DNA complexes are labeled with a gray triangle. The 12% native polyacrylamide gel was used for analysis. The white triangles represent the shift bands of unbound DNA fragments. The gray triangles represent the shift bands of DNA fragments (P8/P9/P10/P11) bound by proteins in medium concentrations same as those of the protein‐bound P3 fragment. The black triangles represent the shift bands of DNA fragments (P8/P9/P10/P11) bound by proteins in high concentrations same as those of the protein‐bound P3 fragment. (C) The binding constants of AlkR to different DNA fragments are distinct. The heat change (upper panel) and the integrated peak areas (lower panel) in the ITC analyses are shown, and the concentrations of DNA fragments and AlkR protein remained constant in the assays.

Furthermore, to determine the precise binding properties of AlkR protein to different regions in the P3 fragment, a series of truncated fragments (P8, P9, P10, and P11 fragments) were generated and Isothermal titration calorimetry (ITC) assays were conducted (Figure 4A).

The results indicated that the interaction of AlkR with the wild‐type P3 fragment adhered to a one‐site binding model, yielding a dissociation constant (*K*_d_) of approximately 0.178 μM. Analysis of the P11 fragment (predicted core binding region –42 to –26 bp from the ATG of the *alkB2* gene) revealed a binding constant of 0.212 μM, also in the low micromolar range, which underscored the strong affinity of AlkR to its specific motif. The interaction stoichiometry of AlkR to the P11 fragment was determined to be 0.224, suggesting that one molecule of the P11 fragment is bound by a tetramer of AlkR (Figure 4B,C, Table S3). In contrast, the binding constants for AlkR with fragments P9 (–50 to –28 bp from the ATG of the *alkB2* gene) and P8 (–27 to –1 bp from the ATG of the *alkB2* gene) were considerably high at 2.06 and 6.70 μM, respectively, reflecting much weaker binding affinities of AlkR to these regions compared to those of P3 and P11 fragments (Figure 4B,C, Table S3). Interestingly, when AlkR protein was mixed with the P9 fragment in an equimolar ratio, a new band shift emerged just under the super‐shift band which was same as that observed with AlkR and the P8 or P11 fragments. However, only a faint band shift was detected with AlkR and the P8 fragment, even at high concentrations (Figure 4B,C). This demonstrates that the principal binding of AlkR to the promoter region of the *alkB2* gene occurs at the specific palindromic motif (5′‐ATTGTCAGACAAT‐3′), with two extra sites in weak binding affinities. The conserved palindromic structure of the core binding motif is essential for efficient DNA binding of AlkR protein.

### The crystal structure insights of AlkR reveal the key residues for its binding to DNA and effector

**Figure 5:**
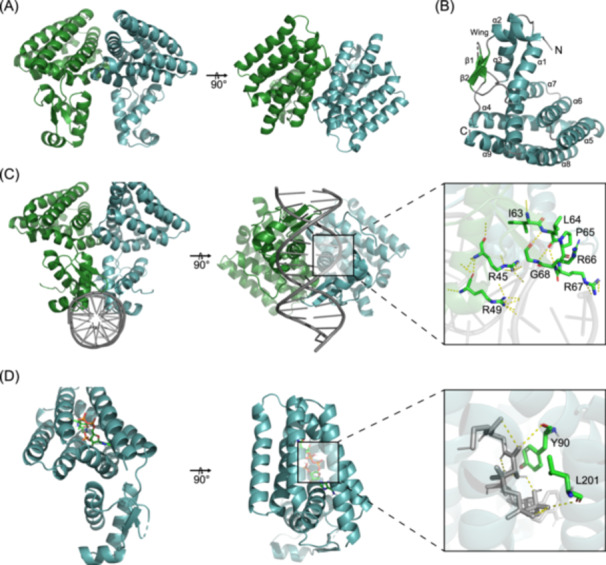
The Apo‐ and holo‐form structures of AlkR protein indicate that AlkR forms homodimers and binds to the specific site of DNA. (A) Top and side views of the homodimers of AlkR protein in Apo form. The data were collected and derived from X‐ray crystallography analysis. (B) Cartoon representation of the AlkR monomer labeled with its secondary structure. (C) AlphaFold 3‐predicted holo‐form structure of DNA‐bound AlkR. The side (left), bottom (middle), and enlarged section (right) views are presented. (D) Predicted holo‐form structure of C18‐CoA‐bound form AlkR. The side (left), top (middle), and enlarged section (right) views predicted by molecular docking are presented.

The crystal of AlkR protein in its Apo form (PDB 9ISW) was obtained with a NaH_2_PO_4_ buffer, and its structure was determined at a resolution of 1.9 Å within the space group I 41 2 2. The asymmetric unit of the structure comprises a dimer of AlkR, with each monomer featuring an N‐terminal DNA binding domain (residues 1–71) and a C‐terminal effector binding domain (residues 76–219) (Figure 5A).

The DNA binding domain of AlkR protein is characterized by the presence of two β‐strands (β1 and β2) and three α‐helices (α1 to α3). The helix–turn–helix motif, formed by α2 and α3, is linked to the anti‐parallel β‐strands (β1 and β2) via a small loop (wing motif). The C‐terminal domain contributes to the secondary structure of AlkR protein with six additional helices (α4 to α9), which are indicative of AlkR protein classification within the VanR‐group regulators belonging to the FCD subfamily of the GntR superfamily (Figure 5A,B). Further, the holo‐form structures of AlkR protein with the palindromic 15‐bp DNA fragment were predicted and generated using AlphaFold 3, illustrating the interaction mode of dimeric AlkR with the specific DNA‐binding motif (Figure 5C). It can be found that each AlkR monomer within the dimer recognizes and binds to a half‐site of the palindromic motif. Also, DNA binding is facilitated by the helix–turn–helix (HTH) motif in the N‐terminal, composed of α‐helices α2 and α3, along with the anti‐parallel β‐strands β1 and β2. These structural elements are all engaged in the polar interactions of AlkR protein with DNA strands.

Normally, positively charged amino acids play an important role in the DNA binding of transcription regulators. On the basis of structure analysis, four arginine residues within AlkR protein (R45, R49, R66, and R67) were identified to be probably crucial for the interaction establishment with the DNA strands. The side chains of these arginine residues are responsible for forming the necessary polar interactions to stabilize the AlkR–DNA complex (Figure 5C). Furthermore, site‐directed mutagenesis of those predicted residues was performed and the mutation effect on DNA binding of AlkR protein was evaluated. The EMSA results indicated that the AlkR mutants of residue R45 or R49 showed significantly diminished DNA‐binding capacities, while those of residue R66 or R67 showed slightly enhanced DNA‐binding abilities (Figure S6A,B,D,E). Structure analysis indicated that residues R45 and R49, situated on the α3 helix of the HTH motif of AlkR protein, can directly form hydrogen bonds with the 15‐bp DNA strands in the major groove. Residues R66 and R67 in the anti‐parallel β‐strands are then engaged in the formation of hydrogen bonds with DNA strands in the minor groove, contributing toward strengthening the DNA binding of AlkR. Besides, residues I63 and G68 are structurally integral to the connecting loop of AlkR protein, and their mutations resulted in a notably decreased affinity of AlkR protein for DNA (Figure S6C,F). Nevertheless, other residues (E80, Y83, V160, and N208) in the adjacent space in this AlkR–DNA complex structure did not seem as important for AlkR binding to its specific motif, due to the negligible influence of their mutations on the DNA‐binding ability (Figure S6G–J). These results demonstrated that the arginine residues in AlkR are crucial for its direct interaction with the specific DNA motif, and other adjacent residues (I63 and G68) can aid binding. It is worth noting that these arginine residues were identically distributed in other AlkR homologs, and the adjacent residues of the arginine residues also showed high conservation (Figure S7). In addition to the conserved residues and the secondary structures, the arginine‐mediated interactions of AlkR homologs with the specific DNA motif are completely consistent in all the AlkR homologs at the tertiary structures (Figure S8). This implies that there exists a universal and conservative DNA‐binding mode in the GntR/VanR transcription regulators.

Moreover, our results showed that C16‐CoA and C18‐CoA could dissociate AlkR protein from its specific DNA motif and facilitate the transcription of the *alkB*2 gene (Figure 2C). The structural modeling results of AlkR protein and C18‐CoA using AutoDock revealed that C18‐CoA was surrounded by the C‐terminal effector‐binding domain of AlkR protein composed of helices α4‒α9. Notably, residues Y90 and L201 were found to interact directly with C18‐CoA (Figure 5D). Among the residues close to C18‐CoA, L82, Y83, L93, F134, F182, and L185 show high conservation in all the AlkR homologs, implying the significance of these residues in the effector binding process of these regulators (Figure S7A). The results of EMSA assays indicated that C18‐CoA of 20 μM could effectively dissociate AlkR protein from its core DNA motif. Further, the site‐mutated proteins of these residues, except Y90 and L185, all slightly promoted the C18‐CoA‐mediated dissociation of AlkR protein from the core DNA motif. The AlkR mutant proteins at L82, F134, or F182 were dissociated by C18‐CoA at a concentration lower than 5 or 10 μM (Figure S9). This suggested that the substitution of the residues close to C18‐CoA into small‐size amino acid residues is likely to promote the function of the effector by facilitating its entry, and this is suitable for the residues potentially interacting with the effector. On the other hand, molecular modeling outcomes of AlkR protein with C12‐CoA and C24‐CoA revealed a lack of compatibility with AlkR protein, suggesting that the release of AlkR protein from the target DNA by the two long‐chain fatty acyl‐CoA (C16‐CoA and C18‐CoA) is influenced by the molecular structure and steric factors (Figure S10). This highly structure‐dependent interaction between AlkR protein and the fatty acyl‐CoA compounds reveals its precise and efficient regulation on the activity of AlkB2 monooxygenase and the utilization of medium‐to‐long‐chain *n*‐alkanes.

### The regulator–enzyme couples of VanR‐AlkB are conserved in *Pseudomonas*

**Figure 6:**
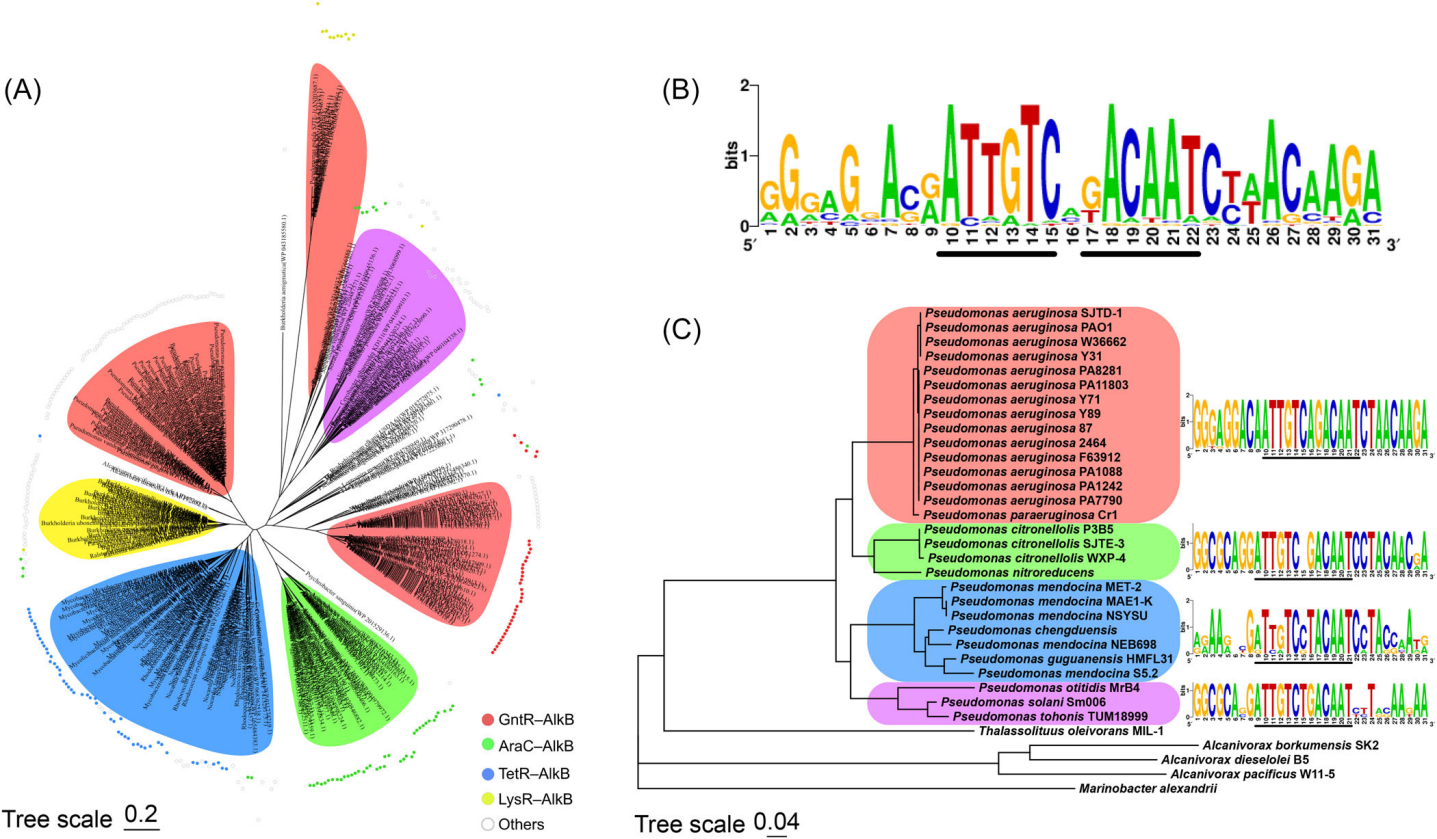
Evolutionary analysis reveals the wide and conservative distribution of VanR–AlkB regulator–enzyme couples. (A) Phylogenetic tree showing that the regulator‐AlkB widely exists in various alkane‐degrading strains. The phylogenetic tree was constructed on the basis of the amino acid sequence of AlkB (309 residues) using the Neighbor‐Joining method. Strains belonging to different families *Pseudomonadaceae* (red), *Acinetobacter* (green), *Mycobacteriaceae* (blue), *Burkholderiaceae* (yellow), and *Rhodobacteraceae* (purple) are labeled with blocks in different colors. The GntR–AlkB (red), AraC–AlkB (green), TetR–AlkB (blue), LysR–AlkB (yellow) couples, and other related proteins (white) are marked by spots in different colors. (B) The conserved core binding sequences of AlkR protein and its homologs. (C) Phylogenetic tree of the GntR–AlkB couples showing that their core binding sites vary in different species. The phylogenetic tree was generated on the basis of the GntR amino acid sequences (left). The core binding sites of different phylogenetic branches were aligned using ClustalW and are shown in a logo graph (right).

In the genome of *P. aeruginosa* SJTD‐1, the *alkR* and *alkB2* genes are oriented in the opposite directions with a 187‐bp intergenic space, encoding the regulator–enzyme couples VanR–AlkB. Bioinformatic analysis showed that similar regulator–enzyme couples are widely distributed across γ‐proteobacteria, suggesting a common and conserved regulatory model for *n*‐alkane utilization (Table S4). The regulators in the couples belong to a variety of transcription factor families, including the GntR family, the AraC family, the TetR family, and the LysR family, and show distinct species‐specific distributions (Figures 6A and S11).

Among them, the GntR family regulators coupled with AlkB monooxygenases are predominant in *Pseudomonadaceae* (29/34), especially in *P. aeruginosa* (13/34), indicating a conserved presence throughout this group of bacteria (Figures 6A and S11). The GntR–AlkB couples are particularly distributed in strains containing two or more AlkB alkane monooxygenases, indicating that at least one AlkB monooxygenase is coupled with a GntR regulator (21/53 in all bacterial groups, 17/17 in *Pseudomonadaceae*). Interestingly, when only one AlkB monooxygenase gene is present in the genome, the associated regulator is invariably a member of the GntR superfamily, demonstrating that GntR regulators play a crucial role in the regulation of AlkB monooxygenases. Further analysis revealed that the GntR regulators in the regulator–enzyme couples in *Pseudomonas* strains all belong to the VanR subclass within the FCD subfamily (Figures 6A and S11); all of them have a similar C‐terminal domain formed by six α‐helices (Figures S7 and S8, Table S4). This highlights that there exists a universal and specific regulation mode for *n*‐alkane utilization in *Pseudomonas* species, potentially with a unique evolutionary trajectory within this genus. In other species, the AraC–AlkB couples are predominantly found in *Acinetobacter* species, typically in the strains containing only a single AlkB monooxygenase. The TetR–AlkB couples are almost exclusively found in *Mycobacteriaceae*, encompassing species such as *Nocardia*, *Mycobacterium*, and *Rhodococcus*. Meanwhile, the LysR–AlkB couples are specifically present in *P. putida*. Even the regulators associated with AlkB monooxygenases varied; the species‐specific distribution of the regulator–enzyme couples suggests a common and unique regulatory model in these bacteria different from others (Figure 6A).

It is worth noting that the high consistency of VanR–AlkB couples was observed not only in the nucleotide sequences of the functional genes but also in the sequences and lengths of the intergenic regions. The interspaces in all the VanR–AlkB couples typically span between 150 bp and 240 bp, and the distinctive core binding motif for AlkR and other VanR regulators (5′‐ATTGTCXXACAAT‐3′) is remarkably conserved across all the intergenic regions. Subtle variations in the sequences of binding motif were found among different species (Figure 6B,C). Interestingly, distinct differences in the core binding sites and the amino acid sequences of VanR regulators along with evolutionary distance were observed in several marine alkane‐degrading strains, including *Thalassolituus oleivorans* MIL‐1, *Marinobacter alexandrii*, *A. borkumensis* SK2, *A. dieselolei* B5, and *A. pacificus* W11‐5 (Figure 6C), while these regulators still show highly similar tertiary structures to those of AlkR and other homologs (Figure S8). The differences may reflect species‐specific adjustment in the regulatory mechanism governing alkane utilization, likely attributable to the early evolutionary separation of microorganisms in different habitats.

In addition, a conserved motif (5′‐GGTAACXCCCTGTGTTACACT‐3′) in the promoter region of *alkR* gene and other regulator genes was aligned in *Pseudomonas* strains with the regulator–enzyme couples. The widespread presence of this motif raises the possibility of self‐regulation for AlkR and other VanR regulators, suggesting that these regulators may also bind to this motif and modulate their own transcription, apart from regulation on AlkB monooxygenases (Figure S12). Moreover, a more in‐depth analysis of the GntR/VanR regulators aligned with the consensus sequence of AlkR through genome databases revealed that almost all of the obtained sequences are coupled with AlkB monooxygenase, particularly in *Pseudomonadaceae* (159/186) and mainly in the strains of *P. aeruginosa* (95/100) and *P. citronellolis* (7/7) (Figure S13). In sum, the collective findings underscore the pivotal role of VanR regulators for precise transcription control of AlkB monooxygenase in the *Pseudomonadaceae* family, which is universal and crucial for quick response and valid utilization of *n*‐alkanes.

## DISCUSSION

**Figure 7:**
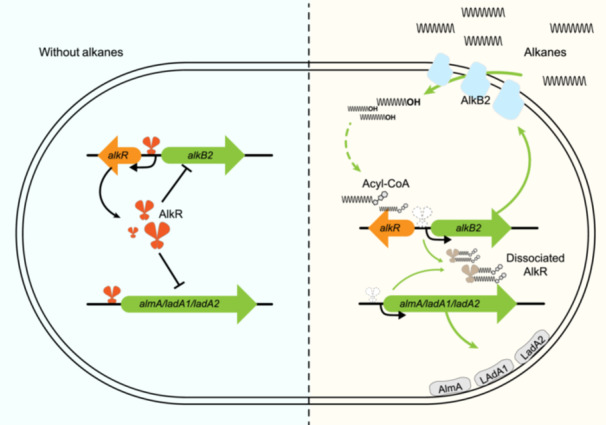
Schematic representation of AlkR regulation of AlkB2 monooxygenase. The AlkR regulation model in response to *n*‐alkane substrates and inducing the transcription of the *alkB2* gene is shown. Left, cells in an environment without alkanes; right, cells in an environment with alkanes.

The AlkB monooxygenases play an important role in the oxidization of *n*‐alkanes among various microorganisms. Hence, rigorous regulation of AlkBs is required to guarantee specific stimulation and precise repression of these enzymes for effective catalyzation and metabolism of different *n*‐alkanes. In this work, a GntR/VanR transcription regulator AlkR in *P. aeruginosa* SJTD‐1 was identified to specifically repress the transcription of AlkB2 monooxygenase and precisely modulate the degradation of medium‐to‐long chain *n*‐alkanes. AlkR protein can recognize and bind to the conservative palindromic motif of the *alkB2* gene and other alkane‐degrading genes (*alm*A, *ladA*1, and *ladA*2) to hinder the transcription. The arginine residues in the structure of AlkR are crucial for DNA interaction, and other adjacent residues can aid this. Moreover, long‐chain fatty acyl‐CoA molecules, specifically C16‐CoA and C18‐CoA, can effectively dissociate AlkR protein and initiate the transcription of the *alkB*2 gene for efficient degradation of medium‐to‐long chain *n*‐alkanes (Figure 7). Bioinformatic analysis demonstrated that the regulatory model of AlkR together with the VanR–AlkB regulator–enzyme couples are distributed widely and conservatively in the *Pseudomonadaceae* family. AlkR stands out as the first GntR/VanR regulator directly involved in the regulation of AlkB monooxygenase and *n*‐alkane utilization. These results can facilitate the study of VanR regulators and illustrate the difference in GntR superfamily members, revealing a conserved regulation pattern of *n*‐alkane utilization.

The GntR superfamily transcription regulators comprise over 93,135 members in the Pfam database, which are characterized by their highly similar N‐terminal HTH domain crucial for DNA binding and their C‐terminal domain for effector binding and oligomerization (E–O). The C‐terminal domain can impose steric constraints on the DNA binding domain and is pivotal for specific and precise regulation of GntR^38^, ^39^. Based on the characteristics of the C‐terminal domains and the secondary structures, the GntR regulators are classified into seven subfamilies, including AraR, DevA, FCD, HutC, MocR, PlmA, and YtrA^40^. Phylogenetic analysis revealed that there are multiple transcription regulators of the GntR superfamily in *P. aeruginosa* SJTD‐1 and AlkR protein is a member of the FCD subfamily, evolutionarily distant from other GntR regulators (Figure S14). The FCD subfamily regulators are divided into two distinct subclasses, FadR and VanR, by the differences in the number of α‐helices (7 α‐helices or 6 α‐helices) in their factor binding domain^40^. FadR subclass regulators are recognized for their vital role in the metabolism of fatty acid, repressing the genes involved in fatty acid degradation via β‐oxidation while simultaneously activating the genes responsible for fatty acid synthesis^41^, ^42^, ^43^. The C‐terminal domains of FadR regulators show variability and long‐chain fatty acyl‐CoA compounds can modulate their binding to DNA^44^, ^45^. In some pathogens, long‐chain fatty fatty acyl‐CoAs, particularly C16‐CoA and C18‐CoA, also serve as crucial effectors of FadR to affect bacterial virulence and motility^46^. In contrast, the VanR subclass regulators are rarely studied, mostly reported to be involved in the regulation of bacterial pathogenesis and virulence^47^, ^48^. Notably, this study is the first to report that AlkR protein is a GntR/VanR regulator involved in the regulation of *n*‐alkane utilization in bacteria.

Furthermore, the FCD subfamily regulators share conserved N‐terminal DNA binding domains, recognizing and binding to short palindromic consensus with a specific sequence (5′‐TGGNNNNNCCA‐3′). Typically, FCD regulators act as a dimer and interact with the symmetric DNA sequences; each monomer recognizes and binds to one half of the palindromic motif^38^, ^39^. Our findings indicated that the specific and core binding motif of AlkR to the promoter region of +the *alkB2* gene in *P. aeruginosa* SJTD‐1 is the palindromic structure motif (5′‐ATTGTCAGACAAT‐3′), similar to the conserved binding motif for FCD regulators. The binding motif of AlkR protein covers the two overlapping ‒10 regions of the *alkB2* gene, capable of playing a regulatory role in diverse environments. Considering that the palindromic structure of the binding motif is more critical for AlkR protein binding than the specific sequences, it is clear that the transcription regulators of the GntR/VanR family share a common mechanism of DNA recognition and binding. Because one transcription regulator may regulate multiple target genes, we further used a palindromic sequence similar to the core binding site of AlkR protein (consisting of 6 nt inverted repeats with a 1 nt spacer) to search in the genome of *P. aeruginosa* SJTD‐1 and investigated the potential targets of AlkR protein (Figure S3). The results showed that several putative sites for AlkR binding were identified in the upstream region of genes associated with the metabolism of fatty acid, glycerolipid, and glycerophospholipid, encoding alkyl hydroperoxide reductase, enoyl‐CoA hydratase, acetyltransferase, thioesterase, the long‐chain fatty acid transporter, alkanesulfonate monooxygenase, glycerol acyltransferase, flippase, lipase, etc (Table S5). Further EMSA assays also demonstrated that AlkR protein could efficiently bind to the promoter regions of *fadD*, *fadBA*, and *fadE* genes, suggesting that AlkR protein may also participate in the regulation of multiple steps in the metabolic pathways of hydrocarbons (Figure S15).

Meanwhile, normally, in bacteria, multiple transcription regulators are involved in the regulation of one enzyme to achieve diverse and flexible control. In strain SJTD‐1, we have found that the distant global regulator CrgA represses the transcription of the *alkB2* gene^36^. The AlkR protein in this work acts as a specific and potent regulator of AlkB2. Deletion of the *crgA* or *alkB* gene alone had negligible impact on the utilization of medium‐to‐long chain *n*‐alkanes by strain SJTD‐1. However, double‐deleted mutants of the two genes resulted in a significant reduction in cell growth, implying the indispensable and complicated regulation for *n*‐alkane utilization (Figure S16). This suggests that although the regulation of CrgA and AlkR to AlkB2 is overlapping to some extent, they may control different pathways and their double deletion adversely affects holistic cell metabolism. Besides, additional deletion of the *crgA* gene (strain *∆alkR∆alkB1∆crgA*) only slightly enhanced the transcription of the *alkB2* gene, while overexpression of the *alkR* gene in this strain caused a significant decrease in the transcription of the *alkB2* gene (Figure S2). Therefore, AlkR plays a dominant regulatory role in the regulation of AlkB2 and the utilization of medium‐to‐long chain *n*‐alkanes in strain SJTD‐1.

Protein recognition of specific DNA sequences is governed by two primary mechanisms, based on the sequence‐dependent deformations of the DNA helix itself or the formation of hydrogen bonds with particular bases predominantly in the major groove of DNA. The latter is dominant in the DNA binding of transcription regulators^49^. Arginine is distinguished by its amphipathic side chain, featuring a positively charged guanidinium group that is highly polar at physiological pH, which can attach to the hydrophobic aliphatic hydrocarbon chain. As a basic amino acid, arginine often distributes on the protein surface and its hydrophilic head group is engaged in critical interactions like hydrogen bonding and salt bridges^50^. In this work, the Apo‐form structure of AlkR dimers revealed that the Arg45 and Arg49 are involved in interactions with the major groove of DNA, by forming hydrogen bonds with specific DNA bases. This interaction is likely pivotal for the recognition and binding of AlkR protein to its specific DNA motif, and removal of the side chains of arginine resulted in the decreased DNA affinity of AlkR protein. Moreover, a flexible sequence (specifically at residues 64–67) in the N‐terminal of AlkR is also important for its DNA binding, predominantly interacting with the minor groove of the DNA helix. Notably, insertion of Arg66 and Arg67 into the minor groove substantially increased the negative electrostatic potential of DNA. This effect is comparable to that observed with the nucleosome core particle and CbpA protein^49^, ^51^. In addition to the vital role of arginine, the role of the scaffold residues is rarely mentioned. Specifically, residues I63 and G68 in AlkR protein are situated on the linker of the flexible minor groove insertion sequence, potentially acting as a scaffold of this region for stabilization by hydrogen bonding via their side chains. The lack of side chains can increase the flexibility of the minor groove insertion region and reduce the DNA binding capacity in turn. It is worth noting that both the DNA‐interacting residues and the scaffold residues conserved in AlkR homologs, forming similar tertiary structures with a specific DNA motif. These findings elucidate the structural basis of GntR/VanR transcription regulators in DNA recognition and binding and indicate their specific and common regulatory mechanism.

As hydrocarbons can serve as a rich nutrient source for microorganisms, they can shape the compositions and structures of microbial communities in enviroments^52^. Microbial strains with diverse temporal and spatial distribution patterns can collaborate in the assimilation and utilization of different hydrocarbon compounds^53^. The genera *Pseudomonas* and *Acinetobacter* have been categorized as R‐strategists due to their rapid growth rates, which enable them to quickly dominate the ecological niches in hydrocarbon‐polluted environments. On the other hand, the genera *Mycobacterium* and *Mycolicibacterium* are considered as K‐strategists. They demonstrate a higher degree of adaptation and stability under the resource‐limited conditions often found in the hydrocarbon‐impacted soils^54^. Hydrocarbons can infiltrate the soil at various depths, and their interaction with the soil composition can selectively enrich distinct microbial populations^55^. For instance, *Rhodococcus* species are enriched at the 20 cm soil depth, while *Acinetobacter* and *Nocardia* species are more prevalent at the 40 cm depth. *Pseudomonas* species have been found to be enriched even at the deeper 80 cm soil layer^56^. This can also be ascribed to the oxygen availability in hydrocarbon‐polluted environments, which is a critical factor for ecological niche occupation. In petroleum‐contaminated soil, the genus *Rhodococcus* predominantly thrives in clear aerobic conditions, while microaerobic conditions favor the overwhelming dominance of the genera *Acinetobacter* and *Pseudomonas* ^57^. This also indicates that there are more vigorous and effective systems for the perceive and metabolism of hydrocarbons in these species, supported by efficient catalyzation and precise regulation.

AlkB monooxygenase is transmembrane metalloenzyme in the microbial degradation of alkanes, catalyzing the initial step in the biodegradation of hydrocarbons. It has significant implications for the bioremediation of petroleum‐polluted environments and is important for global carbon cycles^18^, ^20^, ^22^. As we showed in this work, regulator–AlkB couples have an evolutionarily conserved distribution in strains of various species, not limited to the alkane‐degrading strains. The VanR–AlkB couples are predominant in strains of the *Pseudomonadaceae* family; the AraC–AlkB and TetR–AlkB couples are widespread in strains of *Acinetobacter* and *Mycobacteriaceae*, respectively. Also, the conservation of the VanR–AlkB pair in *Alcanivorax* species suggests that this regulatory pattern for *n*‐alkane degradation may be more broadly present across species not only in the *Pseudomonadaceae* family. This means that the divergence of the regulator–AlkB couples underscores the universal catalytic mode for hydrocarbon utilization and the distinct evolutionary paths of the species‐specific regulatory mode, probably related to the fitness and characteristics of the microorganisms in various ecosystems. The diversity of regulator–AlkB couples and the ecological occupation of different species may interact as both cause and effect.

The *Pseudomonadaceae* family is known for its high adaptability in various stress environments and excellent utilization capability of diverse carbon sources, and accounts for the majority of alkane degraders^58^. *Pseudomonadaceae* strains possess multiple alkane monooxygenases for valid utilization of *n*‐alkanes; therefore, at least with one regulator–AlkB couple, all of the regulators in are VanR regulators in all *P. aeruginosa* strains. This suggests that VanR regulators may serve as biomarkers to identify strains with high alkane‐utilizing ability and as targets to improve the efficiency of alkane‐degrading strains. These characteristics of *Pseudomonas* strains also account for their high prevalence in hydrocarbon‐contaminated environments, highlighting their significant application potential in the removal of pollutants and bioremediation of polluted environments. Moreover, in the marine‐origin strains, we found that there are at least two *alkB* genes in *Alcanivorax* strains and one is the VanR–AlkB pair, which may be associated with their robust capacities in *n*‐alkane degradation^32^, ^59^. *Thalassolituus oleivorans* is known as a obligate utilization species of *n*‐alkanes; however, *Marinobacter alexandrii* has been rarely reported in *n*‐alkane degradation^60^, ^61^. This finding can also provide new insights for potential applications of *Marinobacter alexandrii* and other marine strains for *n*‐alkane degradation and hydrocarbons utilization in ocean.

## MATERIALS AND METHODS

### Bacterial strains and growth conditions

The bacterial strains and plasmids used in this study are listed in Table S1. All *Escherichia coli* strains were grown in Luria–Bertani (LB) medium (Tryptone, 10 g/l; Yeast extract, 5 g/l; NaCl, 8 g/l; pH 7.2) at 37°C. *P. aeruginosa* SJTD‐1 and its derivative strains were cultivated at 37°C in LB medium or minimal medium (KH_2_PO_4_, 4.5 g/l; K_2_HPO_4_·3H_2_O, 13.75 g/l; MgSO_4_·7H_2_O, 0.16 g/l; (NH_4_)_2_SO_4_, 2.0 g/l; FeSO_4_, 5 μg/l; CaCl_2_·2H_2_O, 11 μg/l; MnCl_2_·4H_2_O, 2 μg/l; pH 7.4) supplemented with *n*‐alkanes of different lengths (C12–C20) or glucose as the sole carbon source. Solid media plates were prepared by adding agar (15.0 g/l) to liquid media. The alkane–hexane solutions were prepared by dissolving *n*‐alkanes with *n*‐hexane (v/v or g/v) to a concentration of 1 g/ml, as *n*‐hexane was neither toxic nor useable for *P. aeruginosa* SJTD‐1^21^. Antibiotics were used at the following concentrations: kanamycin, 50 μg/l; ampicillin, 100 μg/l; and gentamycin, 50 μg/l. The cell densities were monitored by measuring culture turbidity at 600 nm (OD_600_). All the chemicals used in this study were purchased from Sigma‐Aldrich.

### Standard DNA manipulation

All oligonucleotides in this study (shown in Table S2) were synthesized by Invitrogen Ltd. DNA polymerases, T4 DNA ligases, and all restriction endonucleases were obtained from TaKaRa Bio Inc and Thermo Fisher Scientific Inc. The heat shock method was used for plasmid transformation into *E. coli* strains, and electroporation was used for plasmid transformation into *P. aeruginosa* strains. The genomic DNAs and plasmid DNAs were extracted using the TIANamp Bacteria DNA Kit and the TIANprep Mini Plasmid Kit, respectively (Tiangen); the amplified PCR fragments were purified using the TIANquick Midi Purification Kit (Tiangen).

### DNA affinity chromatography and mass spectrometry analysis

DNA affinity chromatography was conducted using the biotin‐magnetic bead according to the manufacturer's protocol (Invitrogen). The fragment of the promoter region of the *alkB*2 gene was amplified using the primers tagged with biotin via an N33‐TEG‐COOH linker (MedChemExpress LLC) and immobilized with streptavidin‐coated magnetic particles. The “protein fishing” assay was performed by incubating the crude protein extracts of *P. aeruginosa* SJTD‐1 cultured in a minimal medium supplied with 500 mg/l C18. The beads were washed three times with washing buffer (50 mM Tris·HCl, 100 mM NaCl, 0.5 mM EDTA, 0.05 mM Triton X‐100, pH 8.0) to remove the nonspecific binding proteins. The bound proteins were eluted with elution buffer (Tris·HCl, 50 mM; NaCl, 1 M; EDTA, 0.5 mM; Triton X‐100, 0.05 mM; pH 8.0) and evaluated by 12% sodium dodecyl sulfate‐polyacrylamide gel electrophoresis (SDS‐PAGE). The protein bands were excised and the N‐terminal amino acid composition was detected by electrospray ionization quadrupole‐time of flight mass spectrometry (ESI‐Q‐TOF).

### Expression and purification of AlkR and its mutant proteins

The fragment of the *alkR* gene was amplified from the genomic DNA of *P. aeruginosa* SJTD‐1 and cloned into plasmid pET28a to generate pET‐alkR, which was transformed into *E. coli* ER2566 for the heterologous expression of AlkR protein. The recombinant strain was grown at 37°C in LB medium with kanamycin to OD_600_ about 0.5 and induced with 1 mM isopropyl‐D‐1‐thiogalactopyranoside (IPTG) at 37°C for 3 h. The cell pellets were collected by centrifugation, re‐suspended with lysis buffer (Na_2_HPO_4_, 50 mM; NaCl, 400 mM; imidazole, 10 mM; phenylmethylsulfonyl fluoride (PMSF), 1 mM; pH 8.0), and treated by sonication. The cell lysate was then centrifuged at 12,000 rpm at 4°C for 30 min. The supernatants were then loaded into a Ni‐NTA column (Bio‐Rad) and washed with washing buffer (Na_2_HPO_4_, 50 mM; NaCl, 400 mM; imidazole, 30 mM; PMSF, 1 mM; pH 8.0). The recombinant AlkR protein was eluted with elution buffer (Na_2_HPO_4_, 50 mM; NaCl, 400 mM; imidazole, 250 mM; PMSF, 1 mM; pH 8.0). The purified AlkR protein was concentrated by ultra‐filtration and visualized by 12% SDS‐PAGE electrophoresis. The protein concentration was determined using the Bradford assay and the purified protein was stored in PBS buffer with 50% glycerol. The *alkR* mutants were cloned, expressed, and purified in the same way. The polymeric state of AlkR protein was analyzed by the addition of ethylene glycol bis (sulfosuccinimidyl succinate) (Sulfo‐EGS) and detected by 8% native PAGE electrophoresis.

### EMSA

EMSA assays were performed to detect interactions of AlkR protein to DNA fragments as described before^36^. Different DNA fragments labeled at the 5′‐terminus withFAM were obtained by PCR amplification from the genomic DNA of strain SJTD‐1 or by direct annealing of the complementary oligonucleotides (10 μM) in the annealing buffer (Tris·HCl, 10 mM; KAc, 50 mM; EDTA, 1 mM; pH 8.0). The annealing mixture was incubated at 95°C for 5 min and then slowly cooled to room temperature. EMSA assays were performed using DNA fragments and AlkR proteins at various protein/DNA ratios in the binding buffer (Tris‐HCl, 20 mM; NaCl, 50 mM; DTT, 1 mM; EDTA, 1 mM; BSA, 0.1 mg/ml; pH 7.5). After incubation at 37°C for 30 min, the mixtures were visualized by native polyacrylamide gels (8% to 12%) on a BioRad Imaging System. The effects of different chemicals (ethyl acetate, acetyl‐CoA, C16, C16‐OH, C16‐COOH, C16‐CoA, C18, C18‐OH, C18‐COOH, C18‐COONa, and C18‐CoA) on the DNA binding of AlkR protein were evaluated by adding these chemicals of different concentrations into the binding system and incubating for 30 min for EMSA detection.

### DNase I foot‐printing assay

To determine the binding motif of AlkR protein in the promoter region of the *alkB*2 gene, the DNase I foot‐printing assay was performed. The 76‐bp fragment in the promoter region of *alkB*2 and AlkR protein were incubated in the binding buffer of 20 µl at 37°C for 30 min. The binding reaction mixture was cooled and incubated at 25°C for 10 min. The DNase I (1 U/µl) was diluted 160 times with dilution buffer (MgCl_2_, 5 mM; CaCl_2_, 0.2 mM), and the diluted enzyme solution of 20 µL was added to the binding reaction mixture for a 2‐min reaction at 25°C. A quenching buffer (SDS, 1%; proteinase K, 0.5 mg/ml) of 8 µl was added immediately and incubated at 65°C for 5 min until DNase I was completely inactivated. The mixture was then loaded onto 15% denatured acrylamide gels, and the gels were visualized using a BioRad Imaging System (Bio‐Rad Laboratories Co., Ltd.). The DNA sequence in the region covered by AlkR protein was determined by sequencing.

### ITC detection

ITC measurements were performed to determine the interaction constants of AlkR protein with different DNA fragments at 25°C using an ITC200 system (MicroCal iTC200, GE). AlkR protein and the target DNA fragments were prepared in PBS buffer with 10% glycerin, followed by filtering and degassing of the solutions. Titration was carried out in a 40 μl syringe containing 50 μM DNA fragments. Each titration consisted of an initial 0.4 μl injection, followed by 20 subsequent injections of 2 μl DNA solutions in 200 μl sample solutions containing 35 μM of AlkR protein in an ITC sample cell, with stirring at 1000 rpm. The stoichiometry (*n*) of the reaction was varied to fit the enthalpy for the reaction (Δ*H*) and the dissociation constant (*K*_d_) with the nonlinear least‐square method using a single‐site binding model in the Origin program for MicroCal ITC version 7.0 (GE).

### Homologous recombination and mutant strain construction

The gene knockout mutants derived from *P. aeruginosa* SJTD‐1 were constructed using the two‐step homologous recombination method^62^. To construct the ∆*alkR* strain, the 500‐bp upstream and the 500‐bp downstream fragments of the *alkR* gene were inserted into plasmid pEX18Gm to generate pEX18Gm‐UD_alkR_. *E. coli* SM10 cells containing plasmid pEX18Gm‐UD_alkR_ were conjugated with strain SJTD‐1 cells, and the mutants were obtained by successively screening on LB plates with gentamycin and LB plates containing 10% sucrose. All the constructed strains were confirmed by PCR amplification and DNA sequencing. Other single‐ or multiple‐gene knockout mutants (*∆alkR*, *∆alkB*1, *∆alkR∆alkB*1, *∆alkR∆alkB*1*∆crgA*) were generated using the same procedures.

### Cell growth and *n*‐alkane degradation detection

Strain SJTD‐1 and its derivate mutants (*∆alkR*, *∆alkB*1, *∆alkR∆alkB*1, *∆alkR∆alkB*1*∆crgA*) were cultured at 37°C for 7 days in minimal medium with 500 mg/l *n*‐alkanes (C14, C16, and C18) as sole carbon sources. The initial cell density was set at OD_600_ of 0.05, and cell density was measured every hour using an Automatic Growth Curve Analyzer. The *n*‐alkane‐degrading efficiencies of different strains were detected every 12 h and analyzed using a GC‐MS system (7890A GC/5975C MS; Agilent) equipped with an Agilent DB‐5 (30 m × 0.25 mm × 0.25 μm; Agilent) gas chromatograph column as previously described^20^.

### RNA extraction, reverse transcription (RT), and quantitative PCR (qPCR)

Strain SJTD‐1 and the mutant strains were inoculated in minimal medium with 0.4% glucose or 500 mg/l *n*‐alkanes (C14, C16, C18) as sole carbon sources and cultured for 3 days. The total RNA was extracted with Total RNA Extraction Reagents (Vazyme) and estimated using a Nanodrop UV spectrophotometer (Thermo Fisher Scientific). Reverse transcription and quantitative PCR were conducted using the gene‐specific primers with the PrimeScript Reverse Transcriptase Kit (TaKaRa) and Premix Ex Taq (Probe qPCR) (TaKaRa) in an IQTM 5 Multicolor Real‐time PCR Detection System (Bio‐Rad Laboratories Co., Ltd). The relative variations in mRNA levels of target genes were normalized to that of the 16S rRNA gene and calculated using the $2^{-\Delta \Delta C_{t}}$ method^63^.

### Measurement of the promoter activity of the *alkB*2 gene

The *egfp* gene was amplified and cloned into plasmid pBST to generate pBSG. The promoter regions of the *alkB*2 gene with or without the upstream *alkR* gene were inserted into the upstream of the *egfp* gene in plasmid pBSG, generating plasmids pBSG‐U_alkB2_ and pBSG‐AlkR‐U_alkB2_. The two plasmids were electroporated into strain SJTD‐1 and mutant S1∆*alkR* and the transformants were cultured in LB medium and the minimal medium with 0.4% glucose or 500 mg/l *n*‐alkanes (C14, C16, and C18). The fluorescence of eGFP protein in different strains was measured by excitation at 485 nm and emission at 527 nm using a fluorescence microplate reader.

### Bioinformatics analysis of AlkR protein

Structure modeling of AlkR proteins with DNA fragments was predicted with AlphaFold 3^64^, and the docking of AlkR protein with effectors were performed with AutoDock Vina^65^. Multiple sequence alignments (MSAs) of DNA sequences and amino acid sequences were performed with the ESPript server^66^. The nucleotide conservation of the *alkB*2 promoter and the intergenic region of *alkR* and *alkB*2 were analyzed using MEME suite software^67^. A phylogenetic tree showing the distribution of AlkBs with or without regulators in Gamma proteobacteria was constructed by MEGA 11 using neighbor‐joining methods and visualized by One Table (tvBOT)^68^, ^69^.

### Crystallization and structure determination of AlkR

Crystallization experiments were performed using the vapor diffusion method with the sitting‐drop technique at 20°C. Drops were equilibrated against a reservoir‐containing well solution of 0.5 ml. The purified AlkR protein aliquots were diluted to 10 mg/ml in a dilution buffer containing 50 mM NaH_2_PO_4_ and 150 mM NaCl (pH 7.5). The apo crystals were obtained by mixing 1 μl of protein with 1 μl of reservoir solution (Potassium thiocyanate, 0.5 M; Bis‐tris propane, 0.1 M; pH 7.0). All the crystals were retrieved, cryogenically protected in the corresponding reservoir solutions containing 10% glycerol, and flash‐frozen in liquid nitrogen for data collection.

The data of X‐ray diffraction were collected in cryogenic conditions at 100 K at beamline BL19U1 (wavelength 0.97852 Å) at the Shanghai Synchrotron Radiation Facility (Shanghai, China) and analyzed using the HKL3000 software package (HKL Research)^70^. The predicted structure (AF‐A0A069Q939‐F1) was used as the search model for molecular replacement in the PHENIX PHASER module^71^. Using PHENIX REFINE, the structure refinement was carried out with 5% random reflections for validating the R‐free value through the refinement process^72^. The COOT was used to manually build and correct the structural models in the refinement rounds^73^. All the refined models were validated in the PDB validation server, and all the figures were generated using PyMOL software (Schrödinger).

## AUTHOR CONTRIBUTIONS

**Wanli Peng**: Conceptualization (equal); data curation (equal); formal analysis (equal); investigation (equal); methodology (equal); writing—original draft (equal); and writing—review and editing (supporting). **Xiuli Wang**: Data curation (equal); formal analysis (equal); investigation (equal); methodology (equal); and writing—original draft (equal). **Qinchen Liu**: Data curation (supporting); investigation (supporting); and methodology (supporting). **Zhihong Xiao**: Data curation (supporting); investigation (supporting); and methodology (supporting). **Fulin Li**: Investigation (supporting) and methodology (supporting). **Nannan Ji**: Formal analysis (supporting); investigation (supporting); and methodology (supporting). **Zhuo Chen**: Investigation (supporting) and methodology (supporting). **Jiaying He**: Investigation (supporting) and methodology (supporting). **Junhao Wang**: Investigation (supporting) and methodology (supporting). **Zixin Deng**: Resources (supporting) and supervision (supporting). **Shuangjun Lin**: Funding acquisition (supporting); resources (supporting); and supervision (supporting). **Rubing Liang**: Formal analysis (lead); funding acquisition (lead); methodology (equal); project administration (lead); supervision (lead); validation (lead); visualization (lead); writing—original draft (equal); and writing—review and editing (lead).

## ETHICS STATEMENT

This article does not contain any studies with human participants performed by any of the authors.

## CONFLICT OF INTERESTS

The authors declare no conflict of interests.

## Supporting information

Supporting information.

Supporting information.

Supporting information.

Supporting information.

Supporting information.

Supporting information.

## Data Availability

The atomic coordinates and structure factors have been deposited at the Protein Data bank under PDB ID 9ISW.

## References

[1] Gaur VK , Gautam K , Sharma P , Gupta P , Dwivedi S , Srivastava JK , et al. Sustainable strategies for combating hydrocarbon pollution: special emphasis on mobil oil bioremediation. Sci Total Environ. 2022;832:155083. 35395309 10.1016/j.scitotenv.2022.155083

[2] Wu B , Guo S , Wang J . Spatial ecological risk assessment for contaminated soil in oiled fields. J Hazard Mater. 2021;403:123984. 33265023 10.1016/j.jhazmat.2020.123984

[3] Ugochukwu UC , Chukwuone NA , Jidere C , Agu C , Kurumeh L , Ezeudu OB . Legacy PAHs in effluent receiving river sediments near a large petroleum products depot in Enugu, Nigeria: human health risks and economic cost of pollution. Environ Pollut. 2022;309:119731. 35820571 10.1016/j.envpol.2022.119731

[4] Yang Y , Zhang ZW , Liu RX , Ju HY , Bian XK , Zhang WZ , et al. Research progress in bioremediation of petroleum pollution. Environ Sci Pollut Res. 2021;28:46877–46893. 10.1007/s11356-021-15310-6 34254241

[5] Varjani SJ . Microbial degradation of petroleum hydrocarbons. Bioresour Technol. 2017;223:277–286. 27789112 10.1016/j.biortech.2016.10.037

[6] Head IM , Jones DM , Röling WFM . Marine microorganisms make a meal of oil. Nat Rev Microbiol. 2006;4:173–182. 16489346 10.1038/nrmicro1348

[7] Rojo F . Degradation of alkanes by bacteria. Environ Microbiol. 2009;11:2477–2490. 19807712 10.1111/j.1462-2920.2009.01948.x

[8] Liu H , Liang R , Tao F , Ma C , Liu Y , Liu X , et al. Genome sequence of *Pseudomonas aeruginosa* strain SJTD‐1, a bacterium capable of degrading long‐chain alkanes and crude oil. J Bacteriol. 2012;194:4783–4784. 22887679 10.1128/JB.01061-12 PMC3415508

[9] Gregson BH , Metodieva G , Metodiev MV , Golyshin PN , McKew BA . Differential protein expression during growth on medium versus long‐chain alkanes in the obligate marine Hydrocarbon‐Degrading bacterium *Thalassolituus oleivorans* MIL‐1. Front Microbiol. 2018;9:3130. 30619200 10.3389/fmicb.2018.03130 PMC6304351

[10] Kang YS , Jung J , Jeon CO , Park W . *Acinetobacter oleivorans* sp. nov. is capable of adhering to and growing on diesel‐oil. J Microbiol. 2011;49:29–34. 21369976 10.1007/s12275-011-0315-y

[11] Zampolli J , Collina E , Lasagni M , Di Gennaro P . Biodegradation of variable‐chain‐length *n*‐alkanes in *Rhodococcus opacus* R7 and the involvement of an alkane hydroxylase system in the metabolism. AMB Express. 2014;4:73. 25401074 10.1186/s13568-014-0073-4 PMC4230829

[12] Feng L , Wang W , Cheng J , Ren Y , Zhao G , Gao C , et al. Genome and proteome of long‐chain alkane degrading *Geobacillus thermodenitrificans* NG80‐2 isolated from a deep‐subsurface oil reservoir. Proc Natl Acad Sci USA. 2007;104:5602–5607. 17372208 10.1073/pnas.0609650104 PMC1838512

[13] Van Hamme JD , Singh A , Ward OP . Recent advances in petroleum microbiology. Microbiol Mol Biol Rev. 2003;67:503–549. 14665675 10.1128/MMBR.67.4.503-549.2003 PMC309048

[14] Naeem U , Qazi MA . Leading edges in bioremediation technologies for removal of petroleum hydrocarbons. Environ Sci Pollut Res. 2020;27:27370–27382. 10.1007/s11356-019-06124-8 31392621

[15] Wang VCC , Maji S , Chen PPY , Lee HK , Yu SSF , Chan SI . Alkane oxidation: methane monooxygenases, related enzymes, and their biomimetics. Chem Rev. 2017;117:8574–8621. 28206744 10.1021/acs.chemrev.6b00624

[16] Smits THM , Balada SB , Witholt B , van Beilen JB . Functional analysis of alkane hydroxylases from gram‐negative and gram‐positive bacteria. J Bacteriol. 2002;184:1733–1742. 11872725 10.1128/JB.184.6.1733-1742.2002 PMC134907

[17] Karthikeyan S , Hatt JK , Kim M , Spain JC , Huettel M , Kostka JE , et al. A novel, divergent alkane monooxygenase (*alkB*) clade involved in crude oil biodegradation. Environ Microbiol Rep. 2021;13:830–840. 34672103 10.1111/1758-2229.13018

[18] van Beilen JB , Funhoff EG . Alkane hydroxylases involved in microbial alkane degradation. Appl Microbiol Biotechnol. 2007;74:13–21. 17216462 10.1007/s00253-006-0748-0

[19] van Beilen JB , Marín MM , Smits THM , Röthlisberger M , Franchini AG , Witholt B , et al. Characterization of two alkane hydroxylase genes from the marine hydrocarbonoclastic bacterium *Alcanivorax borkumensis* . Environ Microbiol. 2004;6:264–273. 14871210 10.1111/j.1462-2920.2004.00567.x

[20] Nie Y , Chi CQ , Fang H , Liang JL , Lu SL , Lai GL , et al. Diverse alkane hydroxylase genes in microorganisms and environments. Sci Rep. 2014;4:4968. 24829093 10.1038/srep04968 PMC4021335

[21] Liu H , Xu J , Liang R , Liu J . Characterization of the medium‐ and long‐chain *n*‐alkanes degrading *Pseudomonas aeruginosa* strain SJTD‐1 and its alkane hydroxylase genes. PLoS One. 2014;9:e105506. 25165808 10.1371/journal.pone.0105506 PMC4148322

[22] Guo X , Zhang J , Han L , Lee J , Williams SC , Forsberg A , et al. Structure and mechanism of the alkane‐oxidizing enzyme AlkB. Nat Commun. 2023;14:2180. 37069165 10.1038/s41467-023-37869-z PMC10110569

[23] Wu RR , Dang Z , Yi XY , Yang C , Lu GN , Guo CL , et al. The effects of nutrient amendment on biodegradation and cytochrome P450 activity of an *n*‐alkane degrading strain of *Burkholderia* sp. GS3. J Hazard Mater. 2011;186:978–983. 21167642 10.1016/j.jhazmat.2010.11.095

[24] van Beilen JB , Funhoff EG , van Loon A , Just A , Kaysser L , Bouza M , et al. Cytochrome P450 alkane hydroxylases of the CYP153 family are common in alkane‐degrading eubacteria lacking integral membrane alkane hydroxylases. Appl Environ Microbiol. 2006;72:59–65. 16391025 10.1128/AEM.72.1.59-65.2006 PMC1352210

[25] Wang W , Shao Z . Diversity of flavin‐binding monooxygenase genes (*almA*) in marine bacteria capable of degradation long‐chain alkanes. FEMS Microbiol Ecol. 2012;80:523–533. 22304419 10.1111/j.1574-6941.2012.01322.x

[26] Shanklin J , Whittle E . Evidence linking the *Pseudomonas oleovorans* alkane ω‐hydroxylase, an integral membrane diiron enzyme, and the fatty acid desaturase family. FEBS Lett. 2003;545:188–192. 12804773 10.1016/s0014-5793(03)00529-5

[27] Nie Y , Liang JL , Fang H , Tang YQ , Wu XL . Characterization of a CYP153 alkane hydroxylase gene in a Gram‐positive *Dietzia* sp. DQ12‐45‐1b and its “team role” with alkW1 in alkane degradation. Appl Microbiol Biotechnol. 2014;98:163–173. 23504079 10.1007/s00253-013-4821-1

[28] Wang W , Wang L , Shao Z . Diversity and abundance of oil‐degrading bacteria and alkane hydroxylase (*alkB*) genes in the subtropical seawater of Xiamen Island. Microb Ecol. 2010;60:429–439. 20683589 10.1007/s00248-010-9724-4

[29] Liu H , Sun WB , Liang RB , Huang L , Hou JL , Liu JH . iTRAQ‐based quantitative proteomic analysis of *Pseudomonas aeruginosa* SJTD‐1: a global response to *n*‐octadecane induced stress. J Proteomics. 2015;123:14–28. 25845586 10.1016/j.jprot.2015.03.034

[30] Kong W , Zhao C , Gao X , Wang L , Tian Q , Liu Y , et al. Characterization and transcriptome analysis of a long‐chain n‐alkane‐degrading strain *Acinetobacter pittii* SW‐1. Int J Environ Res Public Health. 2021;18:6365. 34208299 10.3390/ijerph18126365 PMC8296198

[31] Kumar S , Zhou J , Li M , Xiang H , Zhao D . Insights into the metabolism pathway and functional genes of long‐chain aliphatic alkane degradation in haloarchaea. Extremophiles. 2020;24:475–483. 32328734 10.1007/s00792-020-01167-z

[32] Wang W , Shao Z . The long‐chain alkane metabolism network of *Alcanivorax dieselolei* . Nat Commun. 2014;5:5755. 25502912 10.1038/ncomms6755

[33] Liang JL , Nie Y , Wang M , Xiong G , Wang YP , Maser E , et al. Regulation of alkane degradation pathway by a TetR family repressor via an autoregulation positive feedback mechanism in a Gram‐positive *Dietzia* bacterium. Mol Microbiol. 2016;99:338–359. 26418273 10.1111/mmi.13232

[34] Liang JL , JiangYang JH , Nie Y , Wu XL . Regulation of the alkane hydroxylase CYP153 gene in a Gram‐positive alkane‐degrading bacterium, *Dietzia* sp. strain DQ12‐45‐1b. Appl Environ Microbiol. 2016;82:608–619. 26567302 10.1128/AEM.02811-15 PMC4711121

[35] Ratajczak A , Geißdörfer W , Hillen W . Expression of alkane hydroxylase from *Acinetobacter* sp. Strain ADP1 is induced by a broad range of *n*‐alkanes and requires the transcriptional activator AlkR. J Bacteriol. 1998;180:5822–5827. 9811637 10.1128/jb.180.22.5822-5827.1998 PMC107653

[36] Ji N , Wang X , Yin C , Peng W , Liang R . CrgA protein represses AlkB2 monooxygenase and regulates the degradation of medium‐to‐long‐chain *n*‐alkanes in *Pseudomonas aeruginosa* SJTD‐1. Front Microbiol. 2019;10:400. 30915046 10.3389/fmicb.2019.00400 PMC6422896

[37] Zhou X , Xing X , Hou J , Liu J . Quantitative proteomics analysis of proteins involved in alkane uptake comparing the profiling of *Pseudomonas aeruginosa* SJTD‐1 in response to *n*‐octadecane and *n*‐hexadecane. PLoS One. 2017;12:e0179842. 28662172 10.1371/journal.pone.0179842 PMC5491041

[38] Hoskisson PA , Rigali S . Chapter 1: variation in form and function the helix‐turn‐helix regulators of the GntR superfamily. Adv Appl Microbiol. 2009;69:1–22. 19729089 10.1016/S0065-2164(09)69001-8

[39] Suvorova IA , Korostelev YD , Gelfand MS . GntR family of bacterial transcription factors and their DNA binding motifs: structure, positioning and co‐evolution. PLoS One. 2015;10:e0132618. 26151451 10.1371/journal.pone.0132618 PMC4494728

[40] Rigali S , Derouaux A , Giannotta F , Dusart J . Subdivision of the helix‐turn‐helix GntR family of bacterial regulators in the FadR, HutC, MocR, and YtrA subfamilies. J Biol Chem. 2002;277:12507–12515. 11756427 10.1074/jbc.M110968200

[41] DiRusso CC , Heimert TL , Metzger AK . Characterization of FadR, a global transcriptional regulator of fatty acid metabolism in *Escherichia coli*. interaction with the fadB promoter is prevented by long chain fatty acyl coenzyme A. J Biol Chem. 1992;267:8685–8691. 1569108

[42] Cronan JE . The *Escherichia coli* FadR transcription factor: too much of a good thing? Mol Microbiol. 2021;115:1080–1085. 33283913 10.1111/mmi.14663 PMC8180525

[43] van Aalten DMF . The structural basis of acyl coenzyme A‐dependent regulation of the transcription factor FadR. EMBO J. 2001;20:2041–2050. 11296236 10.1093/emboj/20.8.2041 PMC125426

[44] Henry M . A new mechanism of transcriptional regulation: release of an activator triggered by small molecule binding. Cell. 1992;70:671–679. 1505031 10.1016/0092-8674(92)90435-f

[45] Shi W , Kovacikova G , Lin W , Taylor RK , Skorupski K , Kull FJ . The 40‐residue insertion in *Vibrio cholerae* FadR facilitates binding of an additional fatty acyl‐CoA ligand. Nat Commun. 2015;6:6032. 25607896 10.1038/ncomms7032 PMC4336772

[46] Pan X , Fan Z , Chen L , Liu C , Bai F , Wei Y , et al. PvrA is a novel regulator that contributes to *Pseudomonas aeruginosa* pathogenesis by controlling bacterial utilization of long chain fatty acids. Nucleic Acids Res. 2020;48:5967–5985. 32406921 10.1093/nar/gkaa377 PMC7293031

[47] Pinheiro J , Lisboa J , Pombinho R , Carvalho F , Carreaux A , Brito C , et al MouR controls the expression of the *Listeria monocytogene*s Agr system and mediates virulence. Nucleic Acids Res. 2018;46:9338–9352. 30011022 10.1093/nar/gky624 PMC6182135

[48] Vigouroux A , Meyer T , Naretto A , Legrand P , Aumont‐Nicaise M , Di Cicco A , et al. Characterization of the first tetrameric transcription factor of the GntR superfamily with allosteric regulation from the bacterial pathogen *Agrobacterium fabrum* . Nucleic Acids Res. 2021;49:529–546. 33313837 10.1093/nar/gkaa1181 PMC7797058

[49] Rohs R , West SM , Sosinsky A , Liu P , Mann RS , Honig B . The role of DNA shape in protein–DNA recognition. Nature. 2009;461:1248–1253. 19865164 10.1038/nature08473 PMC2793086

[50] Santra S , Jana M . Influence of aqueous Arginine solution on regulating conformational stability and hydration properties of the secondary structural segments of a protein at elevated temperatures: a molecular dynamics study. J Phys Chem B. 2022;126:1462–1476. 35147426 10.1021/acs.jpcb.1c09583

[51] Chintakayala K , Sellars LE , Singh SS , Shahapure R , Westerlaken I , Meyer AS , et al. DNA recognition by *Escherichia coli* CbpA protein requires a conserved arginine–minor‐groove interaction. Nucleic Acids Res. 2015;43:2282–2292. 25670677 10.1093/nar/gkv012 PMC4344490

[52] Cabral L , Giovanella P , Pellizzer EP , Teramoto EH , Kiang CH , Sette LD . Microbial communities in petroleum‐contaminated sites: structure and metabolisms. Chemosphere. 2022;286:131752. 34426136 10.1016/j.chemosphere.2021.131752

[53] Wang A , Fu W , Feng Y , Liu Z , Song D . Synergetic effects of microbial‐phytoremediation reshape microbial communities and improve degradation of petroleum contaminants. J Hazard Mater. 2022;429:128396. 35236043 10.1016/j.jhazmat.2022.128396

[54] Juteau P , Rho D , Larocque R , LeDuy A . Analysis of the relative abundance of different types of bacteria capable of toluene degradation in a compost biofilter. Appl Microbiol Biotechnol. 1999;52:863–868. 10616721 10.1007/s002530051604

[55] Mitter EK , Germida JJ , de Freitas JR . Impact of diesel and biodiesel contamination on soil microbial community activity and structure. Sci Rep. 2021;11:10856. 34035323 10.1038/s41598-021-89637-y PMC8149423

[56] Chaîneau CH , Morel J , Dupont J , Bury E , Oudot J . Comparison of the fuel oil biodegradation potential of hydrocarbon‐assimilating microorganisms isolated from a temperate agricultural soil. Sci Total Environ. 1999;227:237–247. 10231986 10.1016/s0048-9697(99)00033-9

[57] Révész F , Figueroa‐Gonzalez PA , Probst AJ , Kriszt B , Banerjee S , Szoboszlay S , et al. Microaerobic conditions caused the overwhelming dominance of *Acinetobacter* spp. and the marginalization of *Rhodococcus* spp. in diesel fuel/crude oil mixture‐amended enrichment cultures. Arch Microbiol. 2019;202:329–342. 31664492 10.1007/s00203-019-01749-2 PMC7012980

[58] Fenibo EO , Selvarajan R , Abia ALK , Matambo T . Medium‐chain alkane biodegradation and its link to some unifying attributes of *alkB* genes diversity. Sci Total Environ. 2023;877:162951. 36948313 10.1016/j.scitotenv.2023.162951

[59] Schneiker S , dos Santos VAM , Bartels D , Bekel T , Brecht M , Buhrmester J , et al. Genome sequence of the ubiquitous hydrocarbon‐degrading marine bacterium *Alcanivorax borkumensis* . Nat Biotechnol. 2006;24:997–1004. 16878126 10.1038/nbt1232 PMC7416663

[60] Yakimov MM , Giuliano L , Denaro R , Crisafi E , Chernikova TN , Abraham WR , et al. *Thalassolituus oleivorans gen. nov*., sp. nov., a novel marine bacterium that obligately utilizes hydrocarbons. Int J Syst Evol Microbiol. 2004;54:141–148. 14742471 10.1099/ijs.0.02424-0

[61] Yang Q , Feng Q , Zhang B , Gao J , Sheng Z , Xue Q , et al. *Marinobacter alexandrii* sp. nov., a novel yellow‐pigmented and algae growth‐promoting bacterium isolated from marine phycosphere microbiota. Antonie Van Leeuwenhoek. 2021;114:709–718. 33751267 10.1007/s10482-021-01551-5

[62] Hoang TT , Karkhoff‐Schweizer RR , Kutchma AJ , Schweizer HP . A broad‐host‐range Flp‐FRT recombination system for site‐specific excision of chromosomally‐located DNA sequences: application for isolation of unmarked *Pseudomonas aeruginosa* mutants. Gene. 1998;212:77–86. 9661666 10.1016/s0378-1119(98)00130-9

[63] Livak KJ , Schmittgen TD . Analysis of relative gene expression data using Real‐Time quantitative PCR and the 2−ΔΔCT method. Methods. 2001;25:402–408. 11846609 10.1006/meth.2001.1262

[64] Abramson J , Adler J , Dunger J , Evans R , Green T , Pritzel A , et al. Accurate structure prediction of biomolecular interactions with AlphaFold 3. Nature. 2024;630:493–500. 38718835 10.1038/s41586-024-07487-w PMC11168924

[65] Eberhardt J , Santos‐Martins D , Tillack AF , Forli S . AutoDock Vina 1.2.0: new docking methods, expanded force field, and python bindings. J Chem Inf Model. 2021;61:3891–3898. 34278794 10.1021/acs.jcim.1c00203 PMC10683950

[66] Robert X , Gouet P . Deciphering key features in protein structures with the new ENDscript server. Nucleic Acids Res. 2014;42:W320–W324. 24753421 10.1093/nar/gku316 PMC4086106

[67] Bailey TL , Johnson J , Grant CE , Noble WS . The MEME suite. Nucleic Acids Res. 2015;43:W39–W49. 25953851 10.1093/nar/gkv416 PMC4489269

[68] Tamura K , Stecher G , Kumar S . MEGA11: molecular evolutionary genetics analysis version 11. Mol Biol Evol. 2021;38:3022–3027. 33892491 10.1093/molbev/msab120 PMC8233496

[69] Xie J , Chen Y , Cai G , Cai R , Hu Z , Wang H . Tree Visualization By One Table (tvBOT): a web application for visualizing, modifying and annotating phylogenetic trees. Nucleic Acids Res. 2023;51:W587–W592. 37144476 10.1093/nar/gkad359 PMC10320113

[70] Otwinowski Z , Minor W . Processing of X‐ray diffraction data collected in oscillation mode. Method Enzymol. 1997;276:307–326. 10.1016/S0076-6879(97)76066-X 27754618

[71] Mccoy AJ , Grosse‐Kunstleve RW , Adams PD , Winn MD , Storoni LC , Read RJ . Phaser crystallographic software. J Appl Crystal. 2007;40:658–674. 10.1107/S0021889807021206 PMC2483472 19461840

[72] Brünger AT . Free R value: a novel statistical quantity for assessing the accuracy of crystal structures. Nature. 1992;355:472–475. 18481394 10.1038/355472a0

[73] Emsley P , Lohkamp B , Scott WG , Cowtan K . Features and development of Coot. Acta Crystallogr D. 2010;66:486–501 20383002 10.1107/S0907444910007493 PMC2852313

