# Supplementary material for: The GntR/VanR transcription regulator AlkR represses AlkB2 monooxygenase expression and regulates n‐alkane degradation in Pseudomonas aeruginosa SJTD‐1
Source: mLife. 2025 Apr 21;4(2):126–42. doi: 10.1002/mlf2.70004 (PMC12042122; doi:10.1002/mlf2.70004)

Fig. S1. The AlkR protein from *P. aeruginosa* SJTD-1 is in dimer form. (A) The SDS-PAGE profile of proteins from the cell lysates of *P. aeruginosa* SJTD-1 enriched by the pull-down assay. The protein marker (M), control group (C), elution solution I (E1) and the elution solution II (E2) were labeled. (B) The SDS-PAGE profiles of the purified recombinant AlkR protein and the EGS cross-linked AlkR protein (EGS) in solution. (C) The FPLC profile for AlkR protein. The elution volume was 14.73 ml. (D) The calibration curve of FPLC generated by using five protein standards.

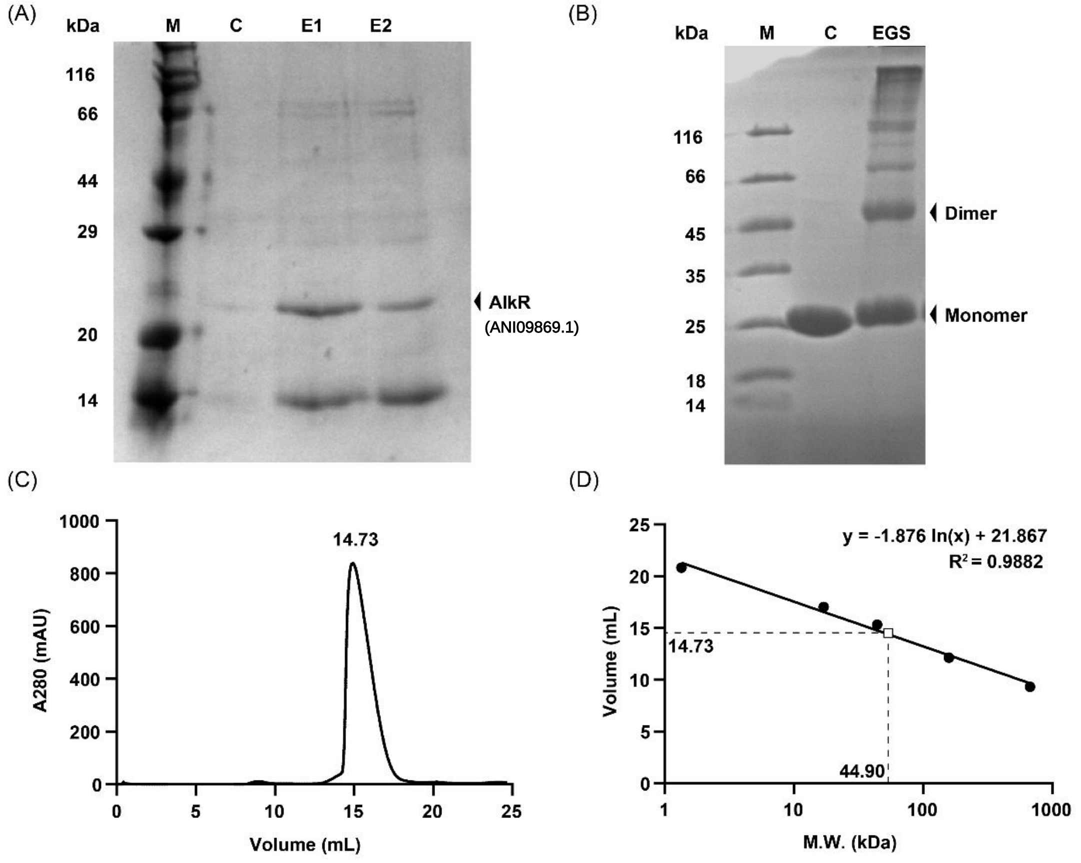

Fig. S2. The transcription of *alkB2* gene is repressed by AlkR and induced by *n*-alkanes (C16 and C18). The strains cultured in medium supplied with 2% glucose were set as control, and all the strains were cultured for 24 h. The transcription levels of *alkB2* gene was detected. The Δ*alkR*Δ*alkB1*Δ*crgA* represented the triple-gene-deleted strain; the Δ*alkR*Δ*alkB1*Δ*crgA*(*alkR*) represented the triple-gene-deleted strain containing the plasmid overexpressing *alkR* gene. Three independent experiments were performed and the average values with standard errors were presented.

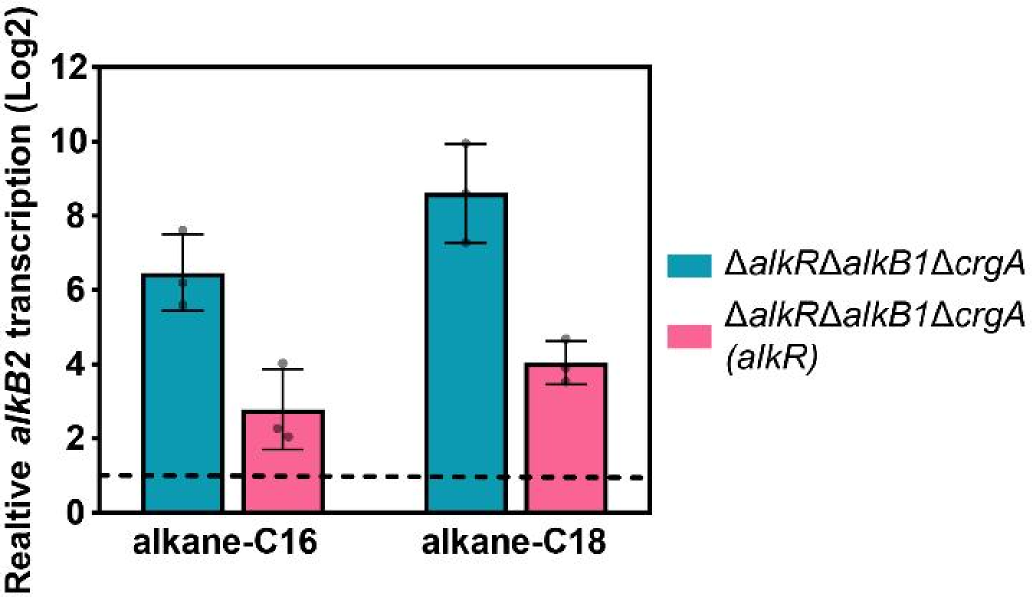

Fig. S3. The expression of AlkR protein can be induced by the medium-to-long chain *n*-alkanes (C14, C16 and C18). The strains cultured in medium supplied with 2% glucose were set as control, and all the strains were cultured for 24 h. The expression of eGFP protein was detected. WT represented the wild-type strain SJTD-1; Δ*alkR* represented the *alkR*-deleted strain; Δ*alkR*(*alkR*) represented the *alkR*-deleted strain containing the plasmid overexpressing *alkR* gene; (*alkR*) represented strain SJTD-1 containing the plasmid overexpressing *alkR* gene. Four independent experiments were performed and the average values with standard errors were presented.

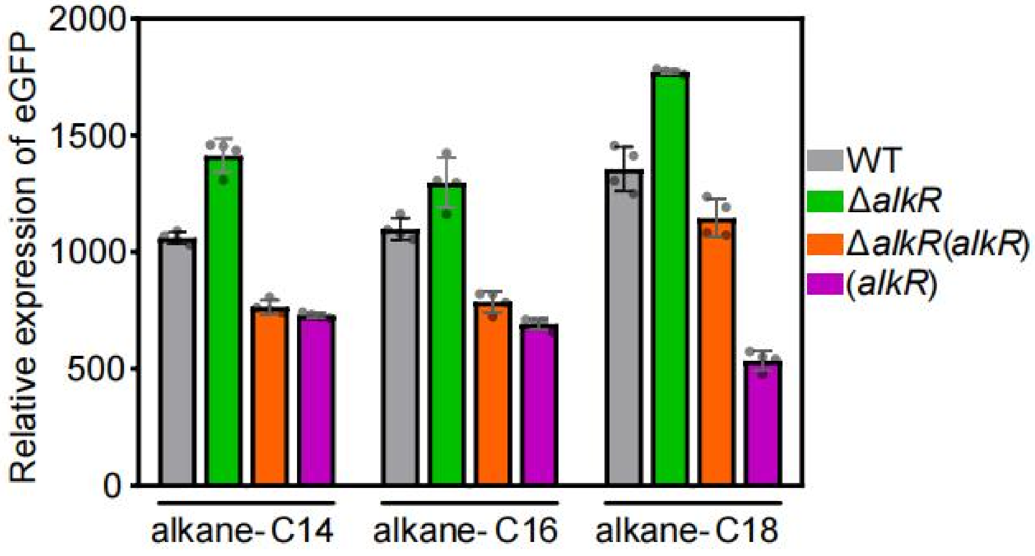

Fig. S4. AlkR protein can bind to the promoter regions of different alkane hydroxylase genes. The 200 bp DNA fragments in the promoter of three alkane hydroxylase genes *almA* (A), *ladA*1 (B), and *ladA*2 (C) were used. The 5'-FAM labeled DNA fragments (3×10^{-8} M) and AlkR (molar ratio 0, 0.2, 1, 2, 10, 20, from left to right) were used. The reaction supplied without protein (-), and reaction supplied with 5×10^{-5} M acety CoA (AC), the C16-CoA, and C18-CoA were used as negative and positive control (molar ratio of protein/DNA was set as 1). The profiles of 8% PAGE from three independent assays were provided. The black triangles reprented the shift bands of unbound DNA fragments (at the bottom) and the shift bands of the protein-bound DNA fragments.

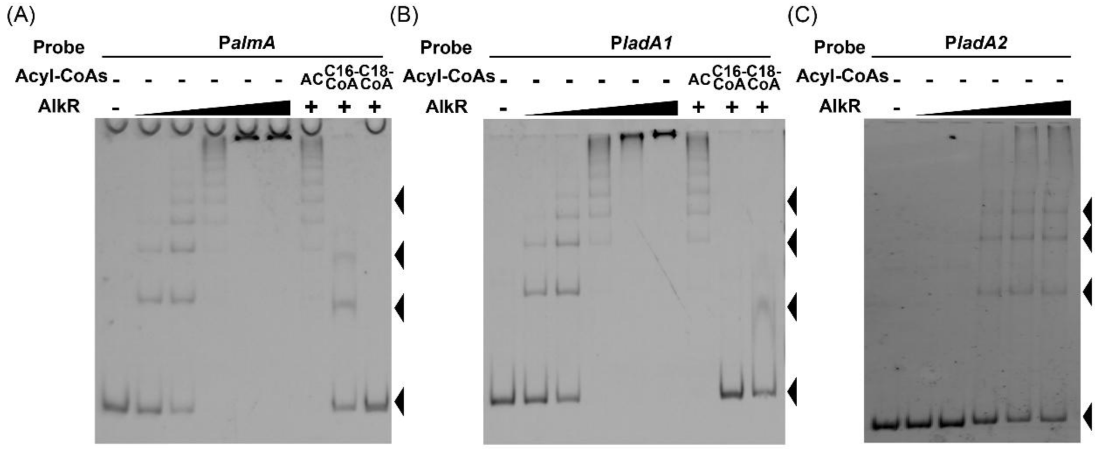

Fig. S5. Only the long-chain fatty acyl-CoA can release the interaction of AlkR with the promoter region of *alkB2* gene. (A) The effect of acetyl-CoA on the interaction of AlkR and the P1 fragment. Different concentrations of acetyl-CoA (150 μM, 300 μM, 600 μM, left to right) were added into the binding system containing AlkR (2.9×10^{-7} M) protein and the P1 fragment (2.8×10^{-8} M), detected by EMSA. (B) The effect of alkane-deriviates on the interaction of AlkR and the P1 fragment. The P1 fragment (2.8×10^{-8} M) and AlkR protein (2.9×10^{-7} M) were mixed, and different effectors (ethyl acetate, C16-alknae, C16-OH, C16-COOH, C16-CoA, C18-alkane, C18-OH, C18-COOH, C18-COONa and C18-CoA) in 600 μM were supplied, detected by EMSA. The profiles of 8% PAGE from three independent assays were presented. The white triangles reprented the shift bands of unbound DNA fragments, and the black triangles reprented the shift bands of the AlkR-bound DNA fragment P1.

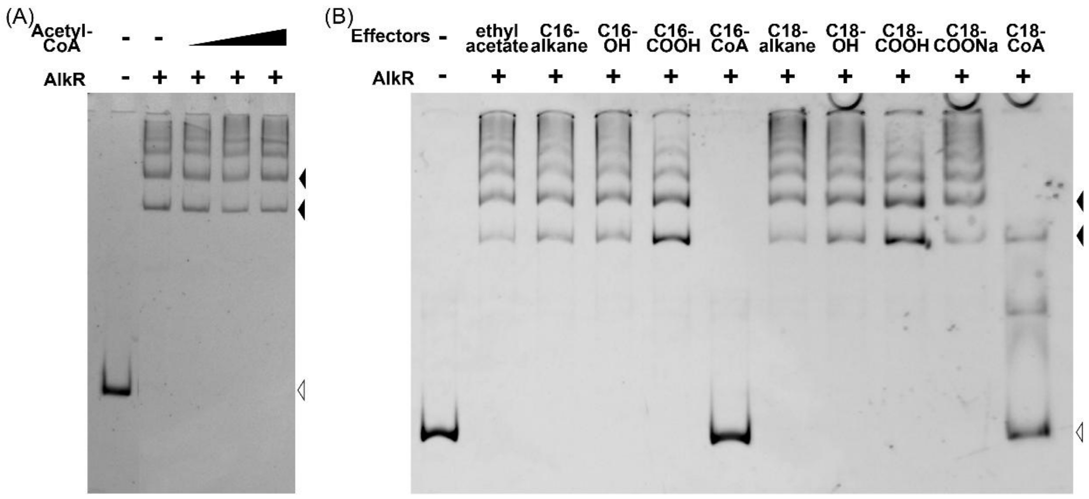

Fig. S6. The binding properties of AlkR mutant proteins to *alkB2* promoter vary. The EMSA assays were used to detect the binding of AlkR mutant proteins to the P11 fragment (2.5 × 10^{-7} M). The molar ratios of AlkR protein/P11 fragment were set as 0.0, 1.0, 2.0, 4.0, 6.0, 8.0, 12.0, 16.0, 20.0 (from left to right). The AlkR mutants R45A (A), R49A (B), I63A (C), R66A (D), R67A (E), G68A (F), E80A (G), Y83A (H), V160A (I), N208A (J), and wild type (K) were used.

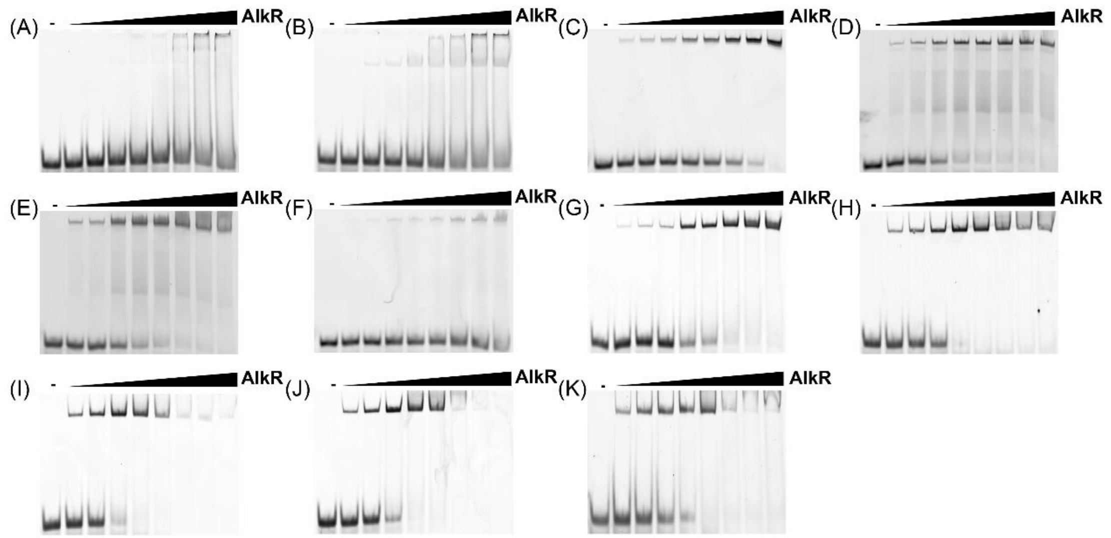

Fig. S7. The multiple sequences alignment (MSA) revealed the conserved sites of AlkR protein and its homologs. (A) The amino acid sequences of AlkR homologous (AXO29833.1 from *P. aeruginosa* Y71, AXL84317.1 from *P. aeruginosa* Y89, AOX40872.1 from *P. aeruginosa* PA11803, AOX34270.1 from *P. aeruginosa* PA8281, APB58329.1 from P. *aeruginosa* PA7790, ASD17112.1 from *P. aeruginosa* Pa1242, AOX27897.1 from *P. aeruginosa* PA1088, ALY46973.1 from *P. aeruginosa* F63912, WRL11614.1 from *P. aeruginosa* 2464, WJQ55528.1 from *P. aeruginosa* 87, AXL72515.1 from *P. aeruginosa* Y31, ALZ18417.1 from *P. aeruginosa* W36662, NP_250217.1 from *P. aeruginosa* PAO1, AVR68609.1 from *P. paraeruginosa* Cr1, ANI15912.1 from *P. citronellolis* SJTE-3, WBG62422.1 from *P. citronellolis* WXP-4, WP_043266695.1 from *P. citronellolis* P3B5, WP_024763910.1 from *P. nitroreducens*, WP_247722438.1 from *P. chengduensis*, AVO51273.1 from *P. mendocina* NEB698, WJH57608.1 from *P. guguanensis* HMFL31, ALN18942.1 from *P. mendocina* S5.2, ATH82574.1 from *P. mendocina* MAE1-K, ARS48621.1 from *P. mendocina* NSYSU, UZZ12735.1 from *P. mendocina* MET-2, BCD87912.1 from *P. solani* Sm006, BCG25653.1 from *P. tohonis* TUM18999, BCA28019.1 from *P. otitidis* MrB, WP_015486341.1 from *Thalassolituus oleivorans* MIL-1, WP_138438935.1 from *Marinobacter alexandrii*, CAL15569.1 from *A. borkumensis* SK2, AFT68391.1 from *A. dieselolei* B5, AJD46447.1 from *A. pacificus* W11-5) from different species were used for multiple sequence analysis. The aligned sequence of the DNA major groove (B) and minor groove (C) insertion site were shown in logo graph.

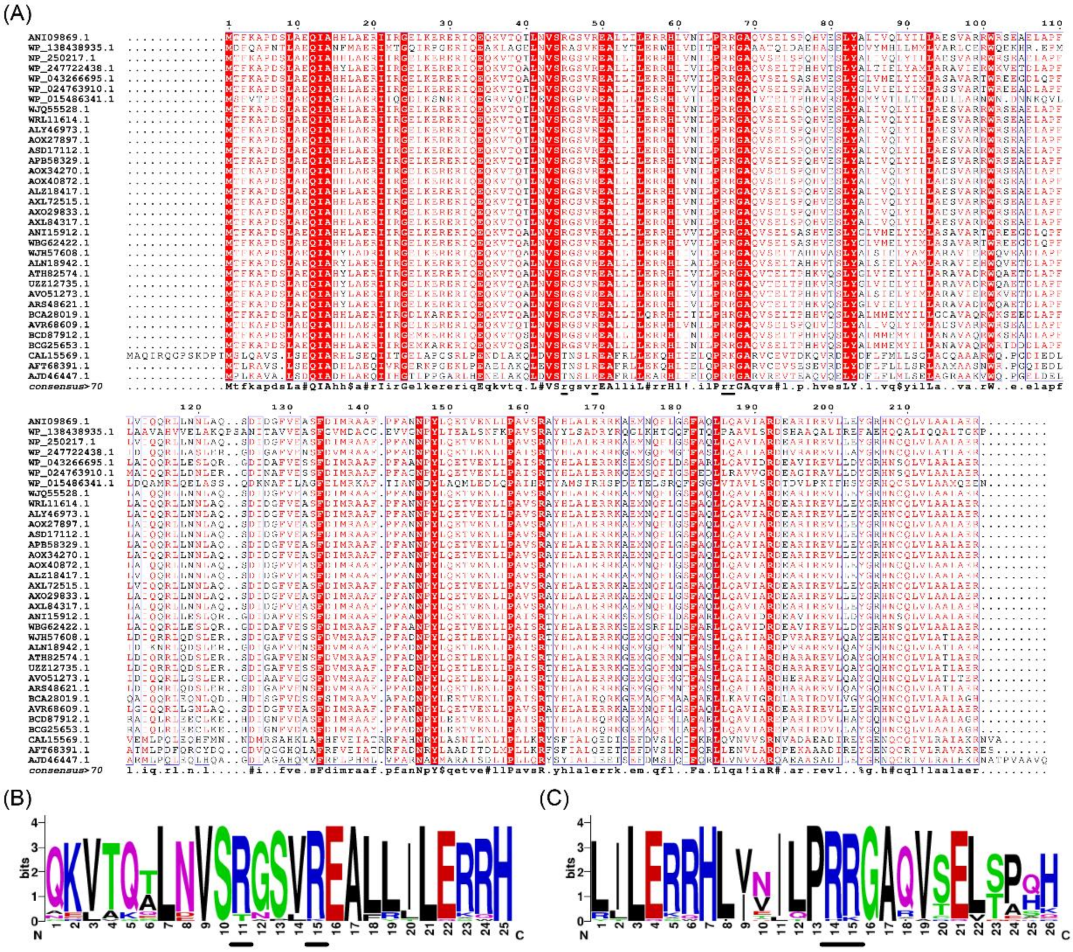
(A)
consensus>70
(B)
(C)
bits
N
C

Fig. S8. The predicted structures of AlkR homologs revealed their conserved DNA binding mode. The DNA binding form of AlkR homologous were predicted with AlphaFold 3 and superimposed (A, B, D, E). The enlarged section of DNA binding sites were showed; the consensus residues of R45, R48, I63, R 66, R67 a nd G68 were labeled (C, F).

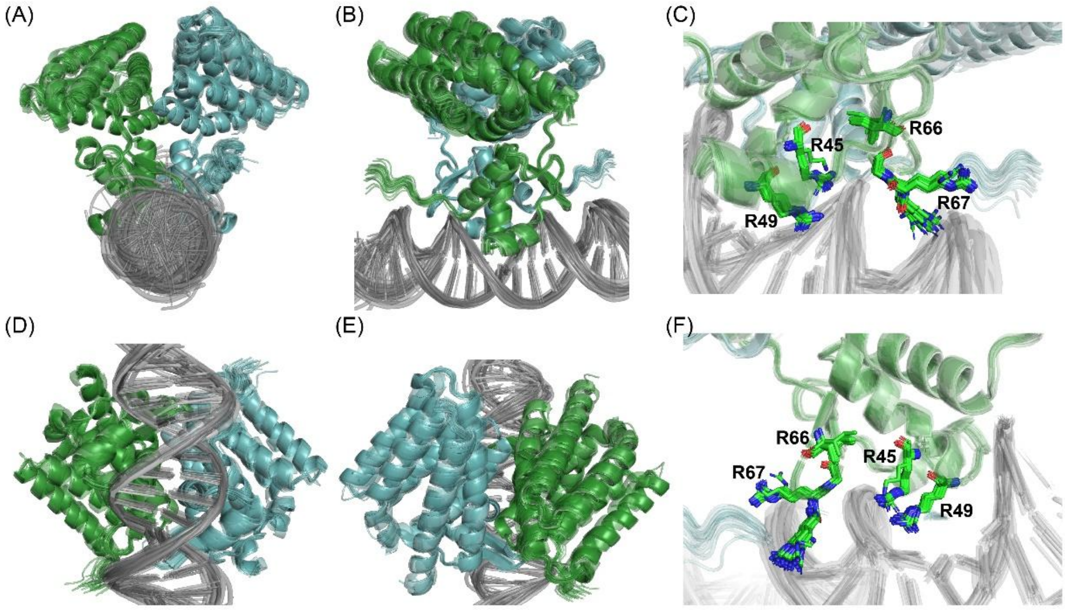

Fig. S9. The releasing properties of AlkR mutants from *alkB2* promoter are discrepant. The EMSA assays were used to detect the releasing effect of C18-CoA on the interaction of AlkR mutants and the P11 fragment (2.5×10^{-7} M). The concentrations of C18-CoA were set as 0.0, 1.0, 2.0, 5.0, 10.0, 20.0, 50.0, 100.0 μM (from left to right). The AlkR mutants L82A (A), Y83A (B), Y90A (C), L93A (D), F134A (E), F182A (F), L185A (G), L201A (H), and wild type (I) were used.

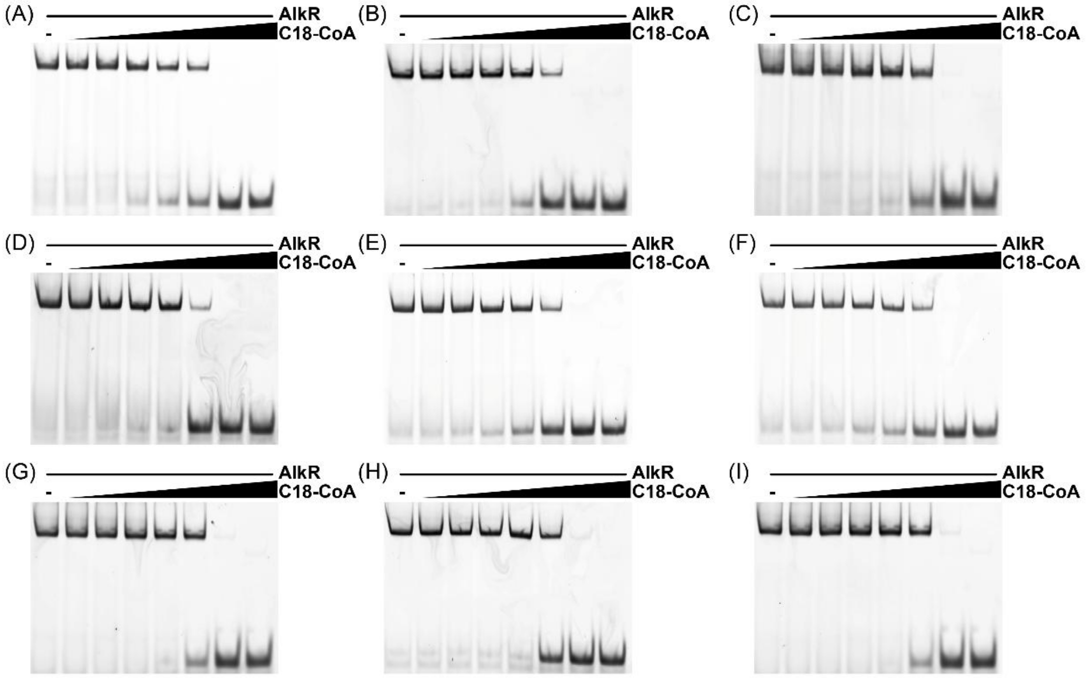

Fig. S10. The predicted binding modes of AlkR to different acteyl CoAs are different. The structures of AlkR binding forms to C12-CoA (A, B, C) and C24-CoA (D, E, F) were predicted with AutoDock. The top (A, D), side (B, E) and enlarged section (C, F) views were shown.

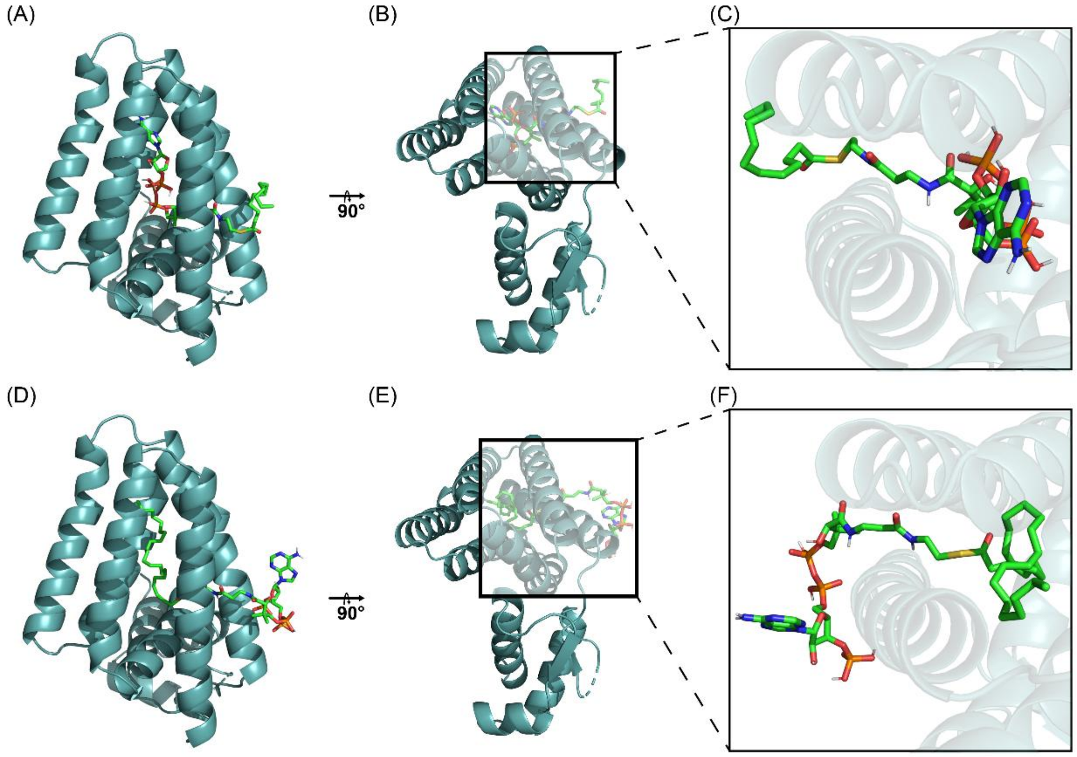

Fig. S11. The phylogenetic tree analysis of the regulator-AlkB couples showed their conservative species-distribution. The phylogenetic tree of various alkanes-degrading strains was constructed on the basis of the amino acid sequences of the AlkB proteins in these strains (309) in the Neighbour-joining method. The GntR-AlkB, AraC-AlkB, TetR-AlkB, LysR-AlkB couples and others were labeled with red, green, blue, yellow and white spots, respectively.

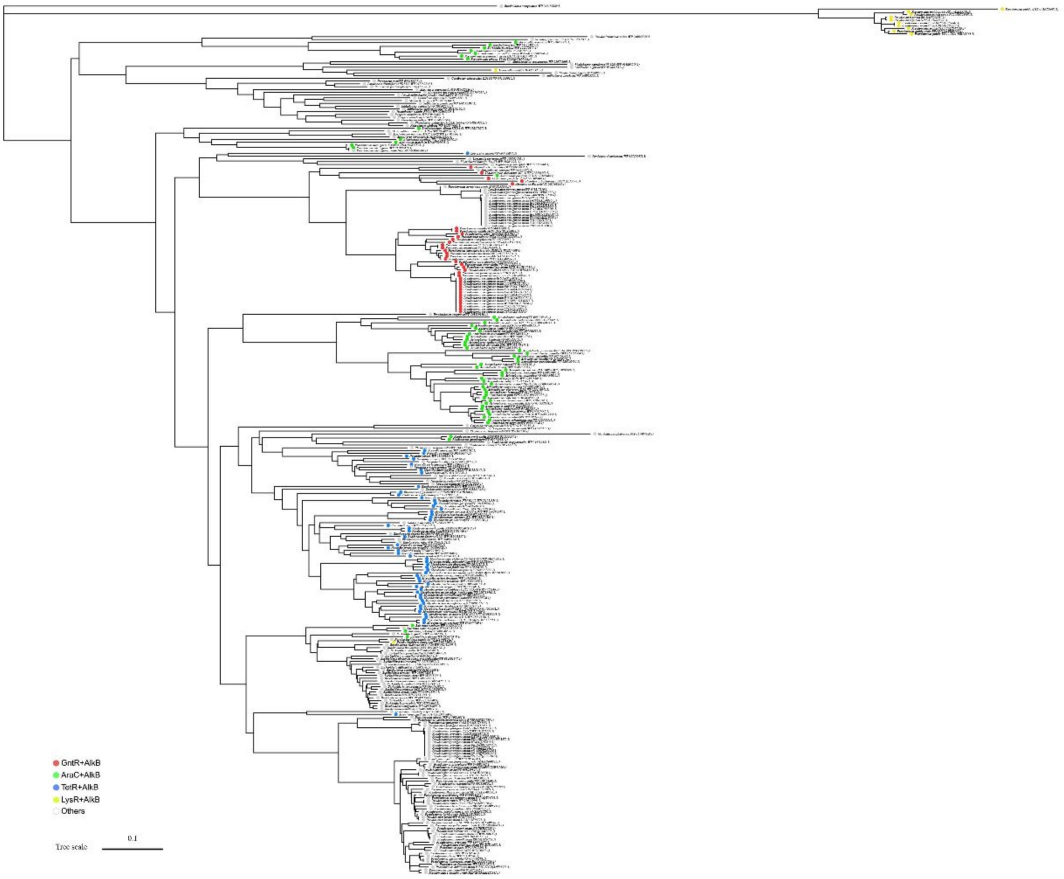

Fig. S12. Multiple sequence analysis (MSA) showed the conserved binding sites in the promoter of AlkR protein and its homologs. (A) The sequences of intergenic promoter region of AlkR homologs from different species of *Pseudomonadaceae* family were aligned; the conserved sites (B) and the core binding sites (C) were shown in logo graph.

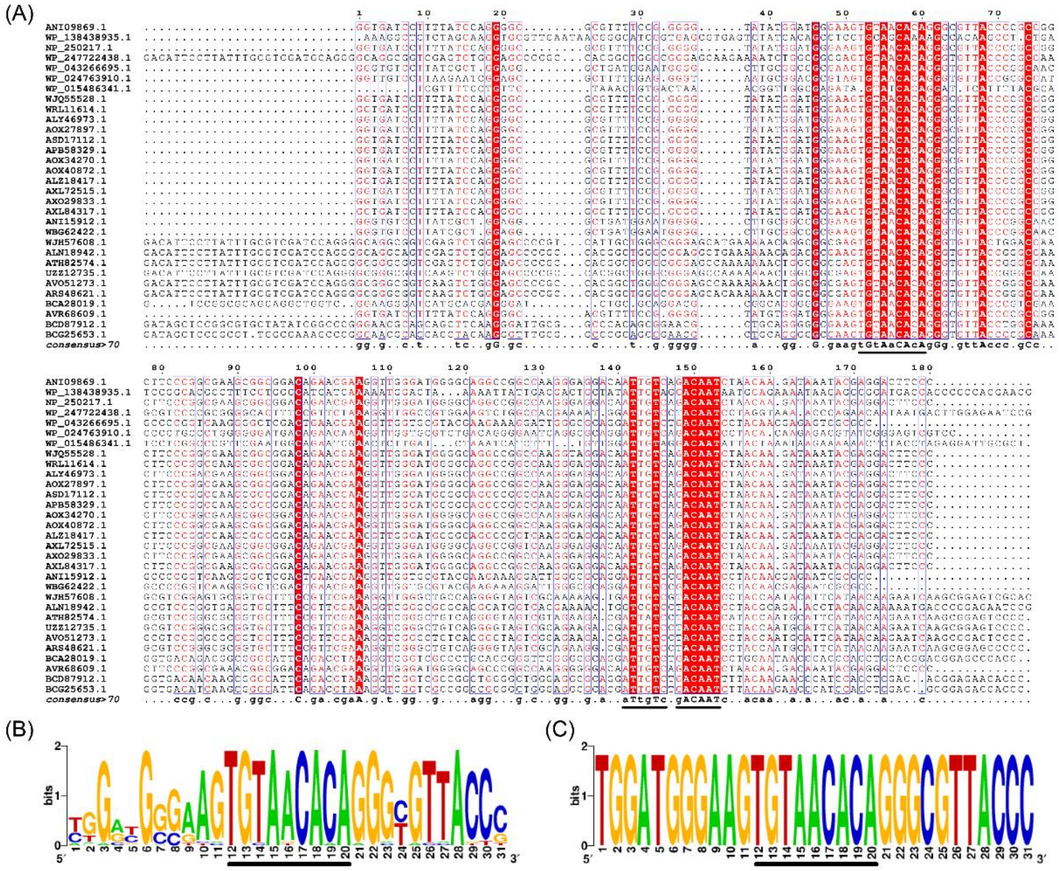

Fig. S13. Phylogenetic analysis of VanR-AlkB couples revealed their conservation in the *Pseudomonadaceae* family. The phylogenetic tree of AlkR homologs from different *Pseudomonas* strains was constructed based on their amino acid sequences in Neighbour-Joining method. *P. aeruginosa* strains were marked with red block; the VanR-AlkB couples were labeled with red spots, and others were labeled with green spots.

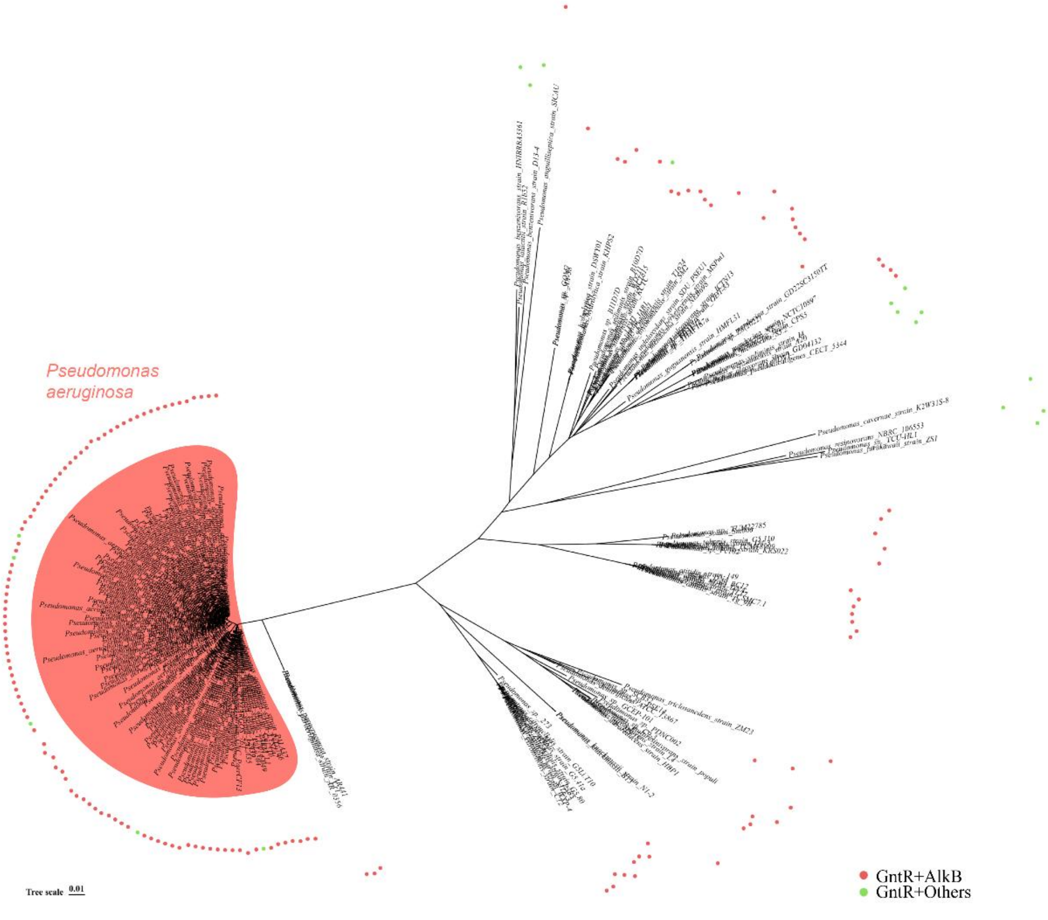

Fig. S14. Phylogenetic analysis of reported GntR superfamily regulators showed the AlkR separation from other FadR members. The reported GntR family regulators from different strains and strain SJTD-1 were analyzed with the MEGA 11, and the phylogenetic tree was constructed based on their amino acid sequences. The bootstrap values were set as 1,000.

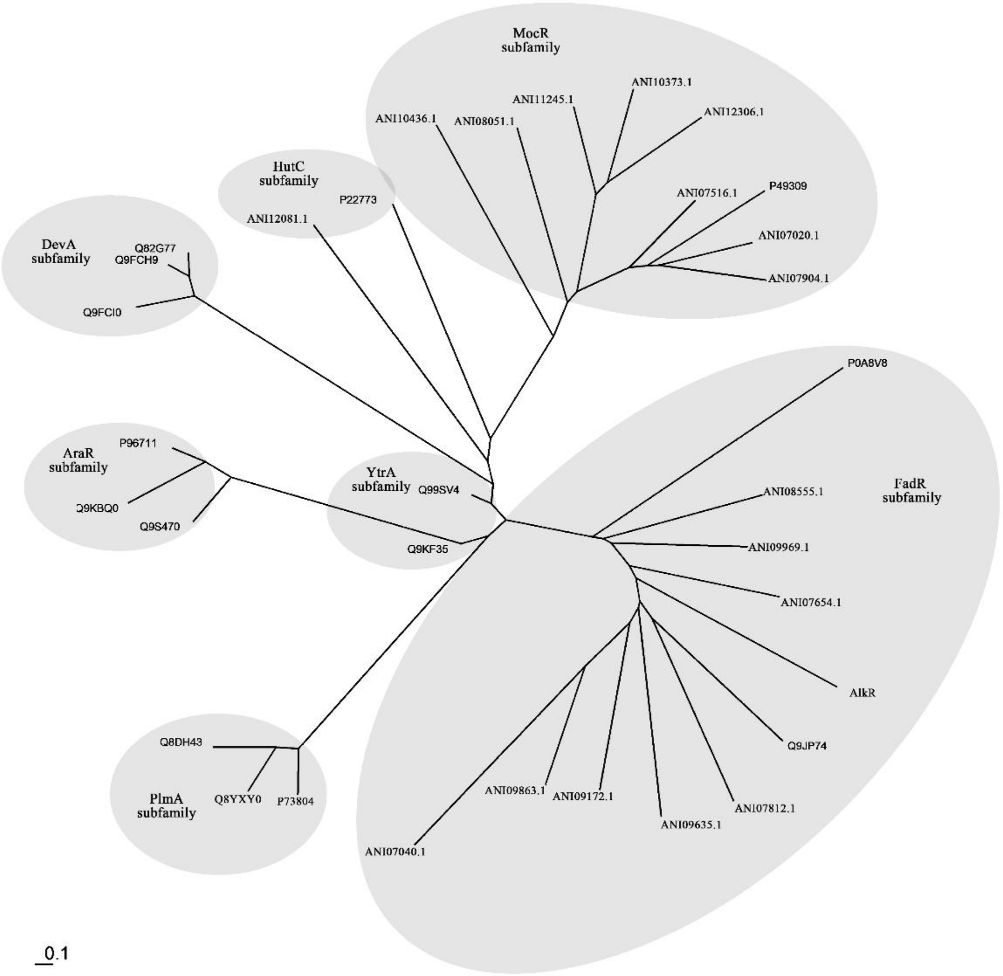

Fig. S15. AlkR protein can recognize and bind to the promoter regions of three genes involved in fatty acid oxidation. The 200 bp DNA fragments in the promoter of three fatty acid oxidation genes *fadD*/*fadBA*/*fadE* were used. The 5'-FAM labeled DNA fragments (3 × 10^{-8} M) and AlkR protein (molar ratio 0, 0.5, 1.0, 2.0, 4.0, 8.0, from left to right) were used. The profiles of 8% PAGE from three independent assays were shown.

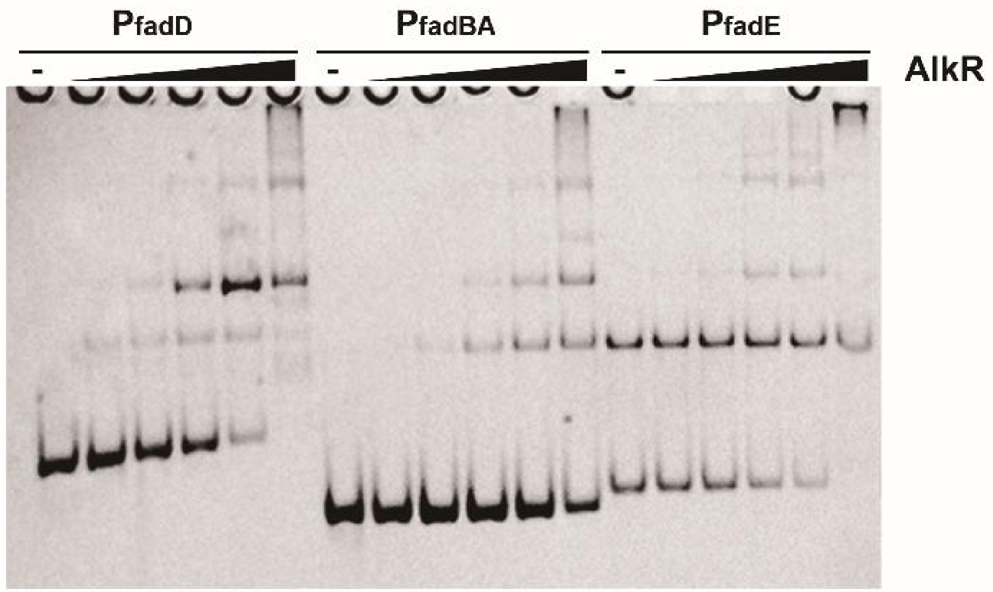

Fig. S16. AlkR protein negatively regulates the alkane-degrading efficiency of *P. aeruginosa* SJTD-1. The alkanes-degrading efficiency and cell growth rate of the two mutant strains (ΔcrgA and ΔalkRΔcrgA) were analyzed in the medium supplied with 500 mg/L of *n*-tetradecane (C14, A), *n*-hexadecane (C16, B), or *n*-octadecane (C18, C). Three independent experiments were performed and the average values with standard errors were presented.

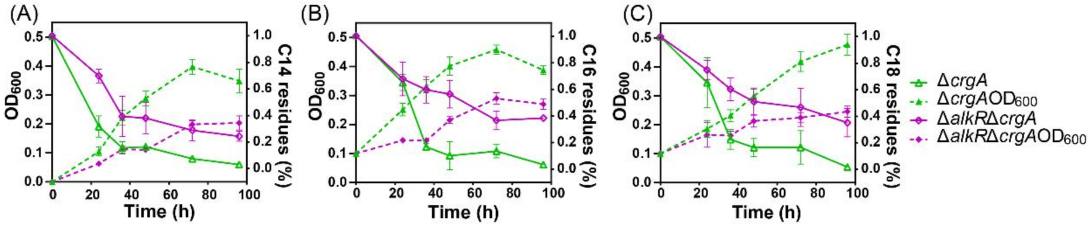

Supplement: Supplementary file 1 — Supporting information. [file MLF2-4-126-s003.pdf]
