# Supplementary material for: The GntR/VanR transcription regulator AlkR represses AlkB2 monooxygenase expression and regulates n‐alkane degradation in Pseudomonas aeruginosa SJTD‐1
Source: mLife. 2025 Apr 21;4(2):126–42. doi: 10.1002/mlf2.70004 (PMC12042122; doi:10.1002/mlf2.70004)
Supplement: Supplementary file 2 — Supporting information. [file MLF2-4-126-s004.docx]

**Table S1 DNA fragments used in this study**

| **DNA fragment** | **Sequence** |
| --- | --- |
| P1 | acgtcatggtgatccttttatccaggggcgcgttttccgggggtatatggatgggaagtgtaacacagggcgttaccccgccggcttcccggcgaagcggcggacagaacgaaggttgggatggggcaggccggccaagggaggacaattgtcagacaatctaacaagataaatacgaggacttccc |
| P2 | aaggttgggatggggcaggccggccaagggaggacaattgtcagacaatctaacaagataaatacgaggacttccc |
| P3 | agggaggacaattgtcagacaatctaacaagataaatacgaggacttccc |
| P4 | agggaggacaattggcaggcaatctaacaagataaatacgaggacttccc |
| P5 | agggaggacaattgttacacaatctaacaagataaatacgaggacttccc |
| P6 | agggaggacaattaccagacaatctaacaagataaatacgaggacttccc |
| P7 | agggaggacaattgtcactaacaagataaatacgaggacttccc |
| P8 | ctaacaagataaatacgaggacttccc |
| P9 | agggaggacaattgtcagacaat |
| P10 | attgtcagacaatctaacaagataaatacgaggacttccc |
| P11 | caattgtcagacaatct |
