# Supplementary material for: The GntR/VanR transcription regulator AlkR represses AlkB2 monooxygenase expression and regulates n‐alkane degradation in Pseudomonas aeruginosa SJTD‐1
Source: mLife. 2025 Apr 21;4(2):126–42. doi: 10.1002/mlf2.70004 (PMC12042122; doi:10.1002/mlf2.70004)
Supplement: Supplementary file 3 — Supporting information. [file MLF2-4-126-s006.docx]

**Table S2 The oligonucleotides used in this study**

| **Names** | | **Sequences (5'-3')*** | **Usages** |  |  |
| --- | --- | --- | --- | --- | --- |
| **Primers for gene cloning** | | |  |  |  |
| alkR-F | | ggggggCATATGACGTTCAAGGCCCCG | Primers used to amplify the *alkR* gene for the construction of plasmid pET-alkR |  |  |
| alkR-R | | ggggggAAGCTTCATCAGCGTTCGGCGAGC |  |  |  |
| egfp-F | | gggcccGGATCCATGGTGAGCAAGGGCGAGGA | Primers used to amplify the *egfp* gene for the construction of plasmid pET-egfp |  |  |
| egfp-R | | gggcccAAGCTTTTAGTACAGCTCGTCCATGCCGAGA |  |  |  |
| alkB2-eF | | gggcccCTCGAGTTCACCGTGGTCGGATCGACG | Primers used to amplify the promoter region of *alkB*2 for the construction of plasmids pBSG-U_alkB2_ and pBSG-AlkR-U_alkB2_ |  |  |
| alkB2-eR | | gggcccAAGCTTGGGAAGTCCTCGTATTTATCTTG |  |  |  |
| alkB2-eF2 | | gggcccCTCGAGGGTGATCCTTTTATCCAG |  |  |  |
| pBS-TF | | gggcccAGGTAGCGAACCCGTCTC | Primers used to amplify framework of plasmid pBSPPc-Gm to delete the T7 and P_c_ promoter and construct plasmid pBST |  |  |
| pBS-TR | | gggcccCTCGAGGGCGTAATAGCGAAGAGGC |  |  |  |
| egfp-eF | | gggcccAAGCTTATGGTGAGCAAGGGCGAGGAG | Primers used to amplify the *egfp* gene for the construction of plasmid pBSG |  |  |
| egfp-eR | | gggcccGGATCCTTAGTACAGCTCGTCCATGCCGAGA |  |  |  |
| egfp-eF2 | | gggcccGAATTCATGGTGAGCAAGGGCGAGGAG | Primers used to amplify the *egfp* gene for the construction of plasmid pBSG-U_alkB2_ |  |  |
| egfp-eR2 | | ggggCCCGGGTTAGTACAGCTCGTCCATGCCGAG |  |  |  |
| **Primers for homologous recombination** | | |  | |  |
| alkB2-UF | gggcccGAATTCTCCGGGGCCTTGAACGTCAT | | Primers used to amplify the upstream fragment of *alkB*2 gene for the deletion of *alkB*2 gene | |  |
| alkB2-UR | gccgcgGATCCGAGCATGCCCAGCAACAGCG | |  |  |  |
| alkB2-DF | gccgcgGATCCTACGAACGCACCACGCCGGA | | Primers used to amplify the downstream fragment of *alkB*2 gene for the deletion of *alkB*2 gene | |  |
| alkB2-DR | gccgcgAAGCTTAGCGGATGCGTTCGGCACCA | |  |  |  |
| alkR-UF | gggcccGAGCTCTGGCCGCGCACGTGTTCG | | Primers used to amplify the upstream fragment of *alkR* gene for the deletion of *alkR* gene | |  |
| alkR-UR | gggcccGTCGACCGGGGCCTTGAACGTCATGGT | |  |  |  |
| alkR-DF | gggcccGTCGACTGATGGCGACCCTGGCGG | | Primers used to amplify the downstream fragment of *alkR* gene for the deletion of *alkR* gene | |  |
| alkR-DR | gggcccAAGCTTCCGGACGCGCCTCGATCA | |  |  |  |
| **Primers for EMSA assay** | | |  | | |
| P1-F | FAM-ACGTCATGGTGATCCTTT | | Primers used to amplify P1 fragment of the promoter region of *alkB*2 gene with 5’-FAM | | |
| P1-R | GGGAAGTCCTCGTATTTATCTTG | |  |  |  |
| P2-F | FAM-AAGGTTGGGATGGGGCAGGCCGGCCAAGGGAGGACAATTGTCAGACAATCTAACAAGATAAATACGAGGACTTCCC | | Primers used to construct the P2 fragment of the promoter region of *alkB*2 gene with 5’-FAM | | |
| P2-R | GGGAAGTCCTCGTATTTATCTTGTTAGATTGTCTGACAATTGTCCTCCCTTGGCCGGCCTGCCCCATCCCAACCTT | |  |  |  |
| P3-F | FAM-AGGGAGGACAATTGTCAGACAATCTAACAAGATAAATACGAGGACTTCCC | | Primers used to construct the P2 fragment of the promoter region of *alkB*2 gene with 5’-FAM | | |
| P3-R | GGGAAGTCCTCGTATTTATCTTGTTAGATTGTCTGACAATTGTCCTCCCT | |  |  |  |
| P8-F | FAM-CTAACAAGATAAATACGAGGACTTCCC | | Primers used to amplify P8 fragment of the promoter region of *alkB*2 gene with 5’-FAM | | |
| P8-R | GGGAAGTCCTCGTATTTATCTTGTTAG | |  |  |  |
| p9-F | FAM-AGGGAGGACAATTGTCAGACAAT | | Primers used to amplify P9 fragment of the promoter region of *alkB*2 gene with 5’-FAM | | |
| p9-R | ATTGTCTGACAATTGTCCTCCCT | |  |  |  |
| P10-F | FAM-ATTGTCAGACAATCTAACAAGATAAATACGAGGACTTCCC | | Primers used to construct P10 fragment of the promoter region of *alkB*2 gene with 5’-FAM | | |
| P10-R | GGGAAGTCCTCGTATTTATCTTGTTAGATTGTCTGACAAT | |  |  |  |
| P11-F | FAM-CAATTGTCAGACAATCT | | Primers used to amplify P11 fragment of the promoter region of *alkB*2 gene with 5’-FAM | | |
| P11-R | AGATTGTCTGACAATTG | |  |  |  |
| P4-F | FAM-AGGGAGGACAATTGGCAGGCAATCTAACAAGATAAATACGAGGACTTCCC | | Primers used to construct P4 fragment of the promoter region of *alkB*2 gene with 5’-FAM | | |
| P4-R | GGGAAGTCCTCGTATTTATCTTGTTAGATTGCCTGCCAATTGTCCTCCCT | |  |  |  |
| P5-F | FAM-AGGGAGGACAATTGTTACACAATCTAACAAGATAAATACGAGGACTTCCC | | Primers used to construct P5 fragment of the promoter region of *alkB*2 gene with 5’-FAM | | |
| P5-R | GGGAAGTCCTCGTATTTATCTTGTTAGATTGTGTAACAATTGTCCTCCCT | |  |  |  |
| P6-F | FAM-AGGGAGGACAATTACCAGACAATCTAACAAGATAAATACGAGGACTTCCC | | Primers used to construct P6 fragment of the promoter region of *alkB*2 gene with 5’-FAM | | |
| P6-R | GGGAAGTCCTCGTATTTATCTTGTTAGATTGTCTGGTAATTGTCCTCCCT | |  |  |  |
| P7-F | FAM-AGGGAGGACAATTGTCACTGTTACTAACAAGATAAATACGAGGACTTCCC | | Primers used to construct P7 fragment of the promoter region of *alkB*2 gene with 5’-FAM | | |
| P7-R | GGGAAGTCCTCGTATTTATCTTGTTAGTAACAGTGACAATTGTCCTCCCT | |  |  |  |
| almA-F | FAM-CATATCGCTCATGTTCCAC | | Primers used to amplify the promoter region of *almA* gene with 5’-FAM | | |
| almA-R | GGCCAAGCTCCTGTTATGATTGTTT | |  |  |  |
| ladA1-F | FAM-CTAAGGGAAAGACTCAAG | | Primers used to amplify the promoter region of *ladA*1 gene with 5’-FAM | | |
| ladA1-R | TCGACTCCGGTGGTTGA | |  |  |  |
| ladA2-F | FAM-ATGGGAGTTCCTTCGACG | | Primers used to amplify the promoter region of *ladA*2 gene with 5’-FAM | | |
| ladA2-R | GTTCGGATAGGTGGCTGGC | |  |  |  |
| fadBA-F | FAM-CGCACCCAAGTCAAACGG | | Primers used to amplify the promoter region of *fadBA* genes with 5’-FAM | | |
| fadBA-R | CAACTGATCTCCACGCTATT | |  |  |  |
| fadD-F | FAM-GCGAAATCGCCTGATAGCCT | | Primers used to amplify the promoter region of *fadD* gene with 5’-FAM | | |
| fadD-R | TGGAAGCCCACTCCTAAGCAAC | |  |  |  |
| fadE-F | FAM-TGGTCACTATCCACGAAGCG | | Primers used to amplify the promoter region of *fadE* gene with 5’-FAM | | |
| fadE-R | CGGAATCCTCCATGAAGACG | |  |  |  |
| **Primers for qPCR detection** | | |  | | |
| 16S-qF | TTCGGACCTCACGCTATCAG | | Primers used for qPCR detection of 16S rDNA gene | | |
| 16S-qR | CATGGCTGGATCAGGCTTT | |  |  |  |
| alkB2-qF | AGGAAGCCAGCGAAGTGCC | | Primers used for qPCR detection of *alkB*2 gene | | |
| alkB2-qR | TCGTGGGAGACGGTGATGC | |  |  |  |

*****The uppercase letters represented the pairing bases, the lowercase letters represented the protecting bases and the underline represented the restriction enzyme sites.
