# Supplementary material for: The GntR/VanR transcription regulator AlkR represses AlkB2 monooxygenase expression and regulates n‐alkane degradation in Pseudomonas aeruginosa SJTD‐1
Source: mLife. 2025 Apr 21;4(2):126–42. doi: 10.1002/mlf2.70004 (PMC12042122; doi:10.1002/mlf2.70004)
Supplement: Supplementary file 4 — Supporting information. [file MLF2-4-126-s002.docx]

**Table S3 The *K_d_* and *N* values of ITC assay of AlkR with different DNA fragments**

| **DNA fragment** | **P3** | **P8** | **P9** | **P10** | **P11** |
| --- | --- | --- | --- | --- | --- |
| ***N* value** | 0.0994 | 0.136 | 0.236 | 0.0990 | 0.224 |
| ***K_d_* value (M^-1^)** | 1.78E7±5.97E6 | 6.70E6±4.18E5 | 2.06E6±3.07E6 | 3.13E6±7.46E5 | 2.12E7±1.64E7 |
